# SecDF as Part of the Sec-Translocase Facilitates Efficient Secretion of *Bacillus cereus* Toxins and Cell Wall-Associated Proteins

**DOI:** 10.1371/journal.pone.0103326

**Published:** 2014-08-01

**Authors:** Aniko Vörös, Roger Simm, Leyla Slamti, Matthew J. McKay, Ida K. Hegna, Christina Nielsen-LeRoux, Karl A. Hassan, Ian T. Paulsen, Didier Lereclus, Ole Andreas Økstad, Mark P. Molloy, Anne-Brit Kolstø

**Affiliations:** 1 Laboratory for Microbial Dynamics (LaMDa), Department of Pharmaceutical Biosciences, School of Pharmacy, University of Oslo, Oslo, Norway; 2 Australian Proteome Analysis Facility (APAF), Macquarie University, Sydney, Australia; 3 INRA, UMR1319 Micalis, Domaine de La Minière, Guyancourt, France; 4 AgroParistech, UMR Micalis, Jouy-en-Josas, France; 5 Department of Chemistry and Biomolecular Sciences, Macquarie University, Sydney, Australia; University of Illinois at Chicago College of Medicine, United States of America

## Abstract

The aim of this study was to explore the role of SecDF in protein secretion in *Bacillus cereus* ATCC 14579 by in-depth characterization of a markerless *secDF* knock out mutant. Deletion of *secDF* resulted in pleiotropic effects characterized by a moderately slower growth rate, aberrant cell morphology, enhanced susceptibility to xenobiotics, reduced virulence and motility. Most toxins, including food poisoning-associated enterotoxins Nhe, Hbl, and cytotoxin K, as well as phospholipase C were less abundant in the secretome of the Δ*secDF* mutant as determined by label-free mass spectrometry. Global transcriptome studies revealed profound transcriptional changes upon deletion of *secDF* indicating cell envelope stress. Interestingly, the addition of glucose enhanced the described phenotypes. This study shows that SecDF is an important part of the Sec-translocase mediating efficient secretion of virulence factors in the Gram-positive opportunistic pathogen *B. cereus*, and further supports the notion that *B. cereus* enterotoxins are secreted by the Sec-system.

## Introduction

*Bacillus cereus sensu stricto* is a Gram-positive spore-forming bacterium producing several toxins associated with food-borne disease. While cereulide has been shown to cause the emetic syndrome [1], the pore-forming toxins cytotoxin K (CytK), haemolysin BL (Hbl) and nonhaemolytic enterotoxin (Nhe) inflict diarrhea [2]–[4]. Fagerlund and co-workers have advocated that secretion of CytK and Nhe- and Hbl-components in *B. cereus* is directed via the Sec-translocase system [5]. SecDF is widely conserved across bacterial genera but is believed to be an accessory, non-essential protein component of the Sec-complex, the main protein secretion machinery in bacteria [6]–[8]. S*ecDF* deletion has been shown to result in low-temperature sensitivity, aberrant cell division and impaired protein secretion in *Escherichia coli*, *Staphylococcus aureus* and *Bacillus subtilis* [9]–[12]. SecDF exhibits the typical structure of RND-type (Resistance-Nodulation-Cell Division) transporters with 12 transmembrane helices and two large extracytoplasmatic loops. However, tertiary and quarternary structures differ from the well described drug efflux-mediating RND transporters. Members of the RND transporter family are generally required for effective efflux of potentially cytotoxic compounds from the cell [13], and their overexpression can confer multi-drug resistance in human pathogens [14]. However, drug efflux is not necessarily the major function of most of the exporters, and their involvement in processes such as metal-ion homeostasis, quorum sensing, maintenance of cell homeostasis, interaction with plant or animal hosts, or efflux of toxic metabolic intermediates, fatty acids or other substances produced by the bacteria themselves, has been reported [15]–[19].

The exact role of SecDF during the protein translocation process has not yet been elucidated in detail. Based on SecDF crystal structures and *in vitro* experiments Tsukazaki and co-workers presented a model describing the proton motive force-dependent role of SecDF during later stage of protein translocation, where efficient protein translocation by SecDF is facilitated by preventing the emerging preprotein from backsliding into the SecYEG channel [20]. Indeed, the charged residues shown to be important for H^+^ translocation by other RND-type transporters are conserved in the SecDF proteins [8]. Interestingly, in an early work Schiebel *et al.* estimated that in the absence of the PMF the costs of protein translocation increase from under 200 ATP units to several thousand ATP molecules per protein [21].

Previous reports suggested that SecDF is not an essential part of the Sec-translocase and fulfills only a noticeable function in secretion under protein hyper-expression and/or low temperature conditions. However, since the protein is ubiquitous, a more profound biological function is plausible. An important role in protein secretion has recently been acknowledged by Quiblier and co-workers ([11], [12], and indeed, a *Staphylococcus aureus secDF* knock out strain displays less virulence in an insect model, and less cytotoxicity to human umbilical vein endothelial cells, than its isogenic wild type strain [12]. In this study we report that SecDF exhibits a substantial function in protein secretion in the spore-forming opportunistic pathogen *B. cereus*, severely affecting cellular export of major toxins and other virulence factors and resulting in reduced virulence of the Δ*secDF* mutant in insect larvae, thus providing additional evidence for Sec-dependent secretion of the *B. cereus* enterotoxins.

## Results

### The ΔsecDF knock out mutant is affected in growth, shape and motility

**Figure 1:**
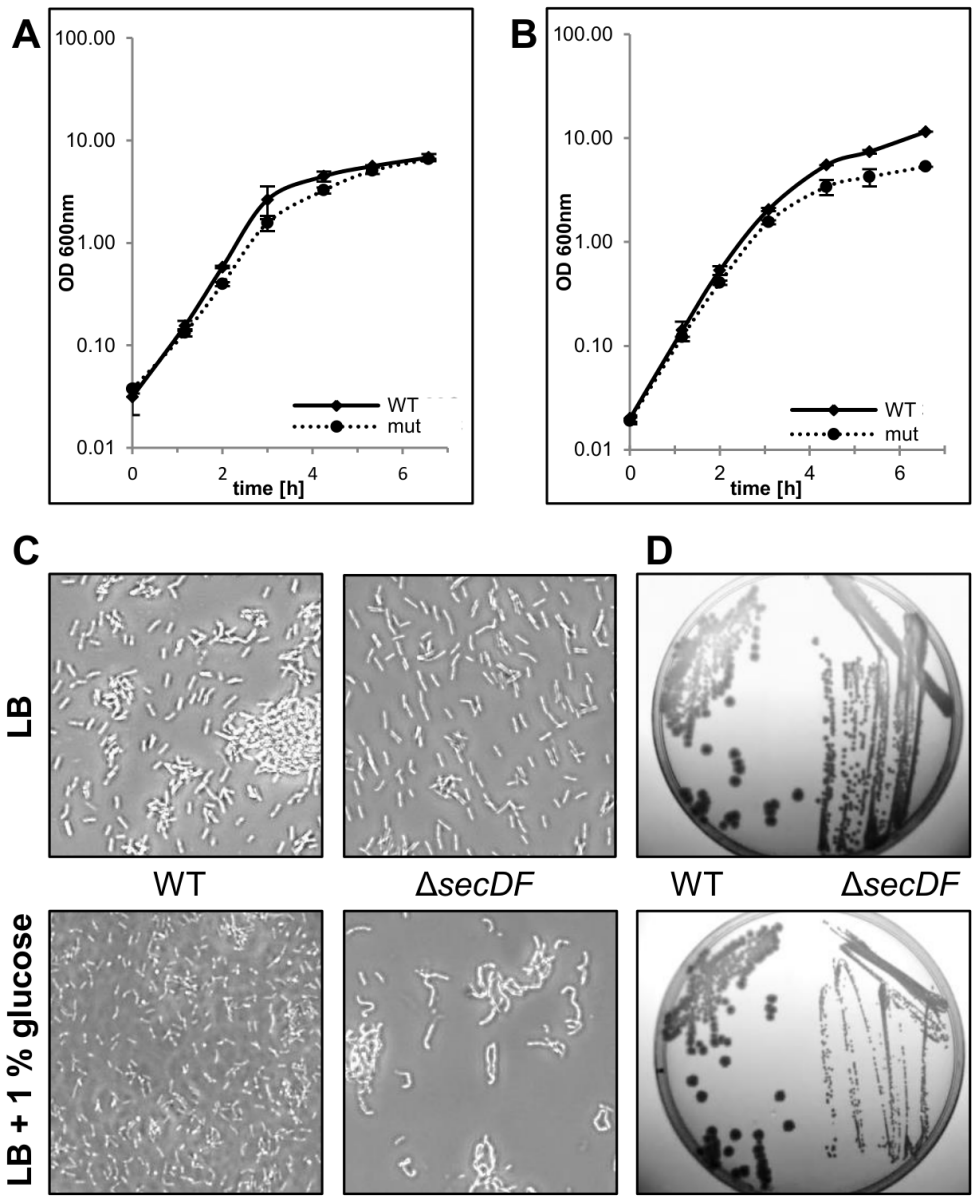
Growth characteristics of the Δ*secDF* mutant in LB with and without glucose. Growth of *B. cereus* ATCC 14579 (WT) and *ΔsecDF* mutant in **A**) LB (no glucose) and **B**) LBG (1% glucose) in shaking cultures at 30°C. The graphs show average OD_600nm_ values with standard deviations of two independent cultures for each strain. **C**) light micrographs of cultures after 24 h growth. **D**) growth of WT (left) and the Δ*secDF* mutant (right) at 30°C for 16 h on LB and LBG agar. All pictures represent results of at least two independent experiments.

A markerless *secDF* deletion mutant was investigated for phenotypic alterations relative to the isogenic wild type strain *B. cereus* ATCC 14579. Bolhuis *et al.* reported a strong activation of the *B. subtilis secDF* promoter by the addition of glucose to the growth medium [9]. There was a small but consistent lag in growth during the exponential phase of Δ*secDF* mutant compared to the wild type in LB medium at 30°C as well as at 37°C (Fig. 1A and data not shown). In LB medium supplemented with 1% glucose (from now on referred to as LBG) growth of the Δ*secDF* mutant was slightly slower than the wild type, and the Δ*secDF* mutant did not reach the culture densities of the wild type at either 20°C, 30°C or 37°C, during the time window investigated (Fig. 1A and data not shown). After 24 h growth, microscopy showed that most Δ*secDF* mutant cells appeared in uncharacteristically crooked chains (Fig. 1B). These growth-related effects of the *secDF* deletion could be circumvented by complementation with SecDF (Fig. S3, left). The mutant displayed a smaller colony size compared to the wild type on LB and LBG agar plates, and this was more pronounced in the presence of glucose (Fig. 1C) and at lower temperatures (data not shown). Growth of the wild type and mutant strains on *B. cereus* agar containing bromothymol blue as pH indicator did not indicate differential production of acidic by-products as a result of glucose fermentation (data not shown).

**Figure 2:**
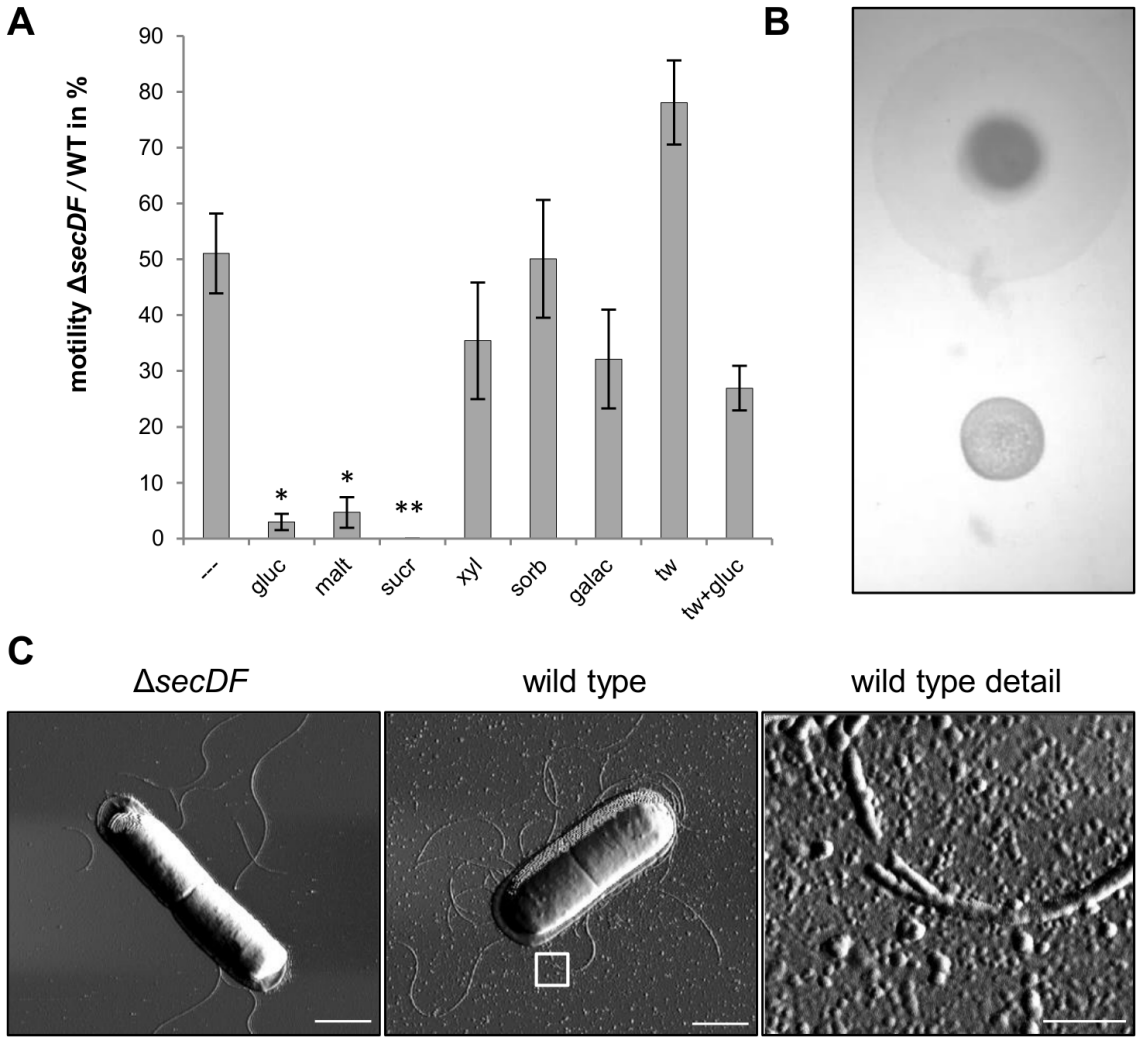
Diminished motility of the Δ*secDF* mutant. **A**) Motility of the Δ*secDF* mutant compared to the wild type strain on 0.3% LB only —, or LB agar plates supplemented with: gluc, 0.4% glucose; malt, 1% maltose; sucr, 1% sucrose; xyl, 1% xylose; sorb, 1% sorbitol; galac, 1% galactose; tw, 0.02% Tween80, tw+gluc, 0.02% Tween80 and 0.4% glucose. The graph shows averages of four to ten independent experiments, error bars represent standard errors and an unpaired Students t-test with two-tailed distribution was performed between wild type and Δ*secDF* mutant (all conditions *P*<0.05). A nonparametric ANOVA with Dunn's multiple comparison *post hoc* test was performed for “LB only” and each of the conditions using additives (*P<0.01; **P<0.001). No movement of the Δ*secDF* mutant was recorded in LB+sucrose in four experiments. **B**) Comparison of motility on 0.7% LBG after 7 h incubation at 30°C; top: wild type; bottom: Δ*secDF* mutant. **C**) AFM amplitude images representative of two independent experiments of cells grown in LBG for 4 h show the grade of flagellation and secretion of putative membrane vesicles. Bars: 1 µm in whole cell images; 0.2 µm in the wild type detail image indicating putative vesicles (arrows).

Microscopy analyses of LBG liquid cultures had clearly showed a decreased motility of the Δ*secDF* mutant compared to the wild type after 4 h of growth. When analyzed on 0.3% LB agar plates, motility of the *ΔsecDF* mutant was approximately half of the wild type, whereas following addition of glucose, maltose or sucrose, the corresponding relative motility was below 10% (Fig. 2A). Severe reduction in motility was also observed on 0.7% LBG agar (Fig. 2B). In *B. subtilis* secretion of the surface-tension reducing compound surfactin enables flagellum-independent motility [22]. To test if differences in surface tension could explain the mutant motility phenotype, Tween 80 was added to the medium [23]. This resulted in partly restored motility of the *secDF* mutant to almost 80% of wild type movement on medium supplemented with Tween 80. Simultaneous addition of Tween 80 and glucose resulted in 75% inhibition of motility relative to wild type under the same conditions (Fig. 2A), showing that a missing surfactant was not the only cause of reduced motility in the *ΔsecDF* mutant. Atomic force microscopy (AFM) amplitude images of Δ*secDF* and wild type cells grown for 4 h in LBG showed that the mutant displayed about five times reduced number of flagella per cell in two independent experiments (Fig. 2C), which may explain its decreased motility (Fig. 2A and B). In addition, AFM amplitude images revealed a higher number of extracellular structures in the wild type compared to the Δ*secDF* mutant samples (Fig. 2C), possibly representing extracellular vesicles [24].

### SecDF deletion reduces resistance of B. cereus to xenobiotics

The 12-transmembrane secondary structure of SecDF is shared by other RND-type transporters known to mediate the efflux of a wide range of xenobiotics. In order to test if SecDF displays similar functions in addition to its role in protein translocation, the effect of SecDF expression in *E. coli ΔacrB* on the susceptibility towards various compounds relative to an empty vector control was tested (Tables S1 and S2). Deletion of *acrB* in *E. coli*, coding for the main xenobiotic efflux transporter in this organism, leads to hypersusceptibility to various toxic compounds [25]. Furthermore, in search for additional phenotypic traits resulting from s*ecDF* deletion in *B. cereus*, minimal inhibitory concentration (MIC) and disk diffusion assays of several xenobiotics were conducted with the *B. cereus* Δs*ecDF* mutant and wild type strains. The Δ*secDF* strain exhibited reduced tolerance to SDS and to the aminoglycoside antibiotic gentamicin, and the reduction in tolerance was amplified in the presence of glucose. We also observed a four-fold decrease in the resistance towards the widely used food preservative sodium benzoate, and a two-fold decreased resistance towards the antimicrobial polymyxin B in LBG medium. Strong effects on growth of the mutant were observed with alcoholic plant extracts of peppermint, calabash plant, and tea tree (Fig. S1). While expression of SecDF from the vector pHT304-pXyl in the wild type *B. cereus* strain did not result in modified resistance to any of the seven compounds tested, heterologous expression of SecDF in *E. coli ΔacrB* produced increased sodium benzoate resistance (Table S2 and data not shown), in accordance with the results from the *B. cereus secDF* deletion mutant.

### The secDF deletion mutant exhibits a reduced level of secreted proteins

**Figure 3:**
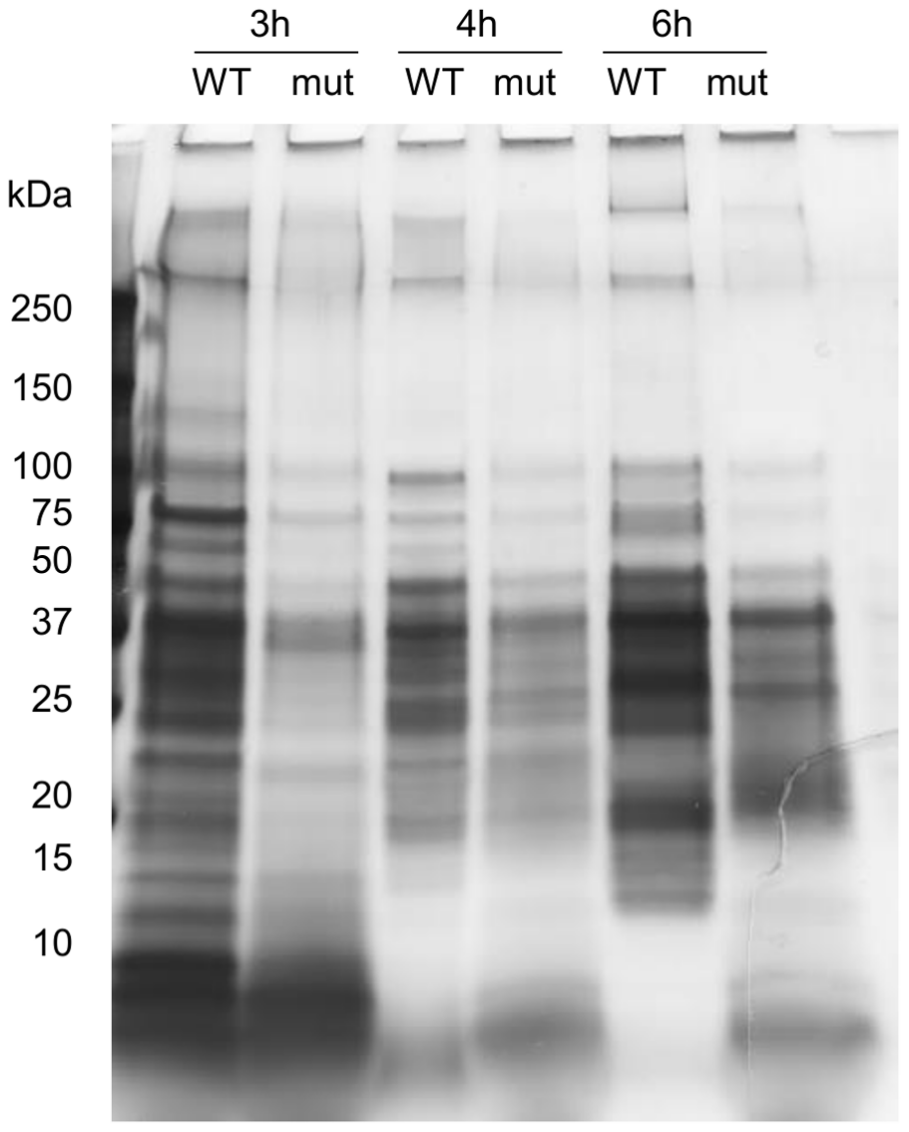
Decreased protein secretion in the Δ*secDF* mutant. The Δ*secDF* mutant secretes less protein than the *B. cereus* ATCC 14579 wild type strain. Equal volumes of normalized and 40-fold concentrated supernatants collected after 3 h (exponential phase), 4 h (transition phase) and 6 h (stationary phase) of growth were applied on 4–20% gradient SDS-PAGE gels and silver stained. The gel represents results of two independent experiments.

To test the effect of deleting *secDF* on the secretome of *B. cereus*, we compared the amount of proteins in the growth medium of the wild type and mutant. Since the phenotypic alterations of the Δ*secDF* mutant seemed to be stronger when grown in glucose-containing medium, secretome analyses were carried out in the presence of 1% glucose. Silver staining following SDS-PAGE revealed a substantial overall reduction of total protein in the growth medium of the Δ*secDF* mutant relative to wild type at different stages of growth (Fig. 3). In addition, an increase of small proteins in the Δ*secDF* mutant secretome was observed. This, however did not seem to be due to an exacerbated proteolytic activity or autolysis rate of the mutant (see method section).

### Label-free mass spectrometry reveals an important role for SecDF in secretion of virulence determinants and putative cell wall modulating enzymes

To further identify which proteins are specifically dependent on SecDF for transport, a label-free proteomic analysis was conducted on three biological replicates of sterile filtered culture supernatants from the Δ*secDF* strain and its respective wild type, after 4 h of growth in LBG medium at 30°C. At this time point, motility of the deletion mutant was visibly reduced and the density of the culture was typically about 65% of the wild type strain (Fig. 1A). In total, 96 proteins were confidently identified in the secretome samples (Table S3). According to the PSORTb algorithm (version 3.0.2; [26]) 29 of these proteins (30%) were either extracellular or cell wall-associated, six (6%) were anticipated to be located within the cytoplasmic membrane, while the majority (55) of the proteins were of cytoplasmic origin (57%). For the remaining 6 proteins (6%) no convincing localization prediction could be made based on sequence similarities with known proteins. However, two of the six contained a putative signal peptide, suggesting an extracellular localization.

Using a paired Students T-test on normalized spectral abundance factors (NSAF, [27]) 34 of the 96 identified proteins were shown to be present at significantly different levels when comparing growth supernatants of the Δ*secDF* mutant and the *B. cereus* wild type (Table 1), indicating fundamental differences in protein secretion between the strains. All the proteins present at reduced levels in the culture supernatant of the Δ*secDF* strain compared to the wild type, were predicted or are known to be extracellular or cell wall-associated (Table 1). Phospholipase C and sphingomyelinase were major protein components in the growth medium of the wild type cells, while they were absent or nearly absent in the *secDF* mutant (Table 1). In addition, the Hbl and Nhe enterotoxin components and cytotoxin K were highly abundant in the extracellular environment of the wild type, while being present at low levels or absent in the mutant secretome. The M9A/M9B – type collagenase C (ColC, BC0556) was 18-fold reduced in the supernatant of the mutant. Another putative collagenase, Sfp (BC3762; also annotated as S-layer protein A), belonging to the intracellular subtilisin-related peptidase S8 group, was identified only in the wild type supernatant, in moderate amounts.

**Table 1:** Proteins found in different amounts in the culture supernatants of the wild type and Δ*secDF* mutant (P-value <0.005).

						WT		Δ*secDF*	
#	Identified Proteins	Localization^1^	locus tag	Uniprot Acc.Nr.	MW (kDa)	NSAF^2^ avg	stdev	NSAF avg	stdev
	**less abundant proteins in the mutant**								
1	Cytotoxin K	EC	BC_1110	Q81GS6	37	**0.14**	0.01	**ND** 3	
2	Enterotoxin/cell-wall binding protein EntB	EC [28]	BC_2952	Q81C32	55	**0.028**	0.004	**0.001**	0.001
3	Perfringolysin O	EC	BC_5101	Q815P0	57	**0.004**	0.001	**ND**	
4	Phospholipase C	EC	BC_0670	Q81HW1	32	**0.55**	0.12	**ND**	
5	Non-hemolytic enterotoxin NheB	EC	BC_1810	Q81EZ7	43	**0.21**	0.05	**0.01**	0.01
6	Putative murein endopeptidase	U	BC_1991	Q81EI5	44	**0.028**	0.007	**ND**	
7	Hemolysin BL lytic component L1	EC	BC_3103	Q7BYC6	44	**0.18**	0.05	**0.01**	0.01
8	Hemolysin BL lytic component L2	EC	BC_3104	Q81BP7	49	**0.25**	0.08	**0.004**	0.003
9	Sphingomyelin phosphodiesterase	EC	BC_0671	Q81HW0	37	**0.28**	0.11	**ND**	
10	putative murein endopeptidase	CW	BC_0991	Q81H34	65	**0.004**	0.001	**ND**	
11	Cell wall endopeptidase, family M23/M37	EC	BC_0740	Q81HR4	42	**0.016**	0.006	**0.001**	0.002
12	Hemolysin BL binding component	EC	BC_3102	Q81BP9	42	**0.10**	0.05	**ND**	
13	Microbial collagenase	EC	BC_0556	Q81I63	109	**0.088**	0.039	**0.005**	0.005
14	Bacillolysin	EC	BC_5351	Q814S1	65	**0.034**	0.013	**0.005**	0.006
15	Non-hemolytic enterotoxin NheA	EC [28]	BC_1809	Q81EZ8	44	**0.12**	0.05	**0.01**	0.01
16	Flagellin*	EC [101]	BC_1657-9	Q81FD3-5	29	**0.51**	0.08	**0.31**	0.10
	**more abundant proteins in the mutant**								
1	DNA-binding protein HU	C	BC_3728	Q81A62	10	**0.15**	0.02	**0.30**	0.02
2	Foldase protein PrsA 1	M	BC_1043	PRSA1_BACCR	32	**ND**		**0.010**	0.002
3	3-oxoacyl-[acyl-carrier-protein] synthase 2	M	BC_1174	Q81GL9	44	**ND**		**0.007**	0.001
4	50S ribosomal protein L10	C	BC_0119	RL10_BACCR	18	**0.009**	0.006	**0.065**	0.011
5	30S ribosomal protein S10	C	BC_0130	RS10_BACCR	12	**0.066**	0.016	**0.183**	0.029
6	DNA-binding protein HU	C	BC_1510	Q81FQ9	12	**0.44**	0.09	**0.75**	0.05
7	Elongation factor G	C	BC_0128	EFG_BACCR	76	**0.041**	0.010	**0.074**	0.005
8	30S ribosomal protein S11	C	BC_0157	RS11_BACCR	14	**0.005**	0.009	**0.077**	0.023
9	Putative triosephosphate isomerase	C	BC_5137	TPIS_BACCR	26	**0.005**	0.009	**0.063**	0.019
10	50S ribosomal protein L6	C	BC_0146	RL6_BACCR	20	**0.002**	0.004	**0.081**	0.028
11	Putative uncharacterized protein	U	BC_p0002	Q814F0	18	**0.10**	0.02	**0.43**	0.12
12	50S ribosomal protein L1	C	BC_0118	RL1_BACCR	25	**0.014**	0.009	**0.065**	0.019
13	50S ribosomal protein L3	C	BC_0131	RL3_BACCR	23	**0.001**	0.002	**0.021**	0.009
14	50S ribosomal protein L15	C	BC_0150	RL15_BACCR	15	**0.003**	0.005	**0.030**	0.011
15	Fructose-bisphosphate aldolase	C	BC_5335	Q814T5	31	**0.027**	0.008	**0.054**	0.010
16	50S ribosomal protein L21	C	BC_4438	RL21_BACCR	11	**0.085**	0.070	**0.225**	0.032
17	50S ribosomal protein L4	C	BC_0132	RL4_BACCR	23	**0.018**	0.007	**0.035**	0.006
18	30S ribosomal protein S15	C	BC_3806	RS15_BACCR	11	**0.022**	0.014	**0.067**	0.022

1 according to prediction of PSORTb algorithm (version 3.0.2; [26]): EC extracellular, C cytoplasmic, U unknown, M membrane; references for experimentally defined locations are given for proteins with predicted unknown localization.

2 **N**ormalized **S**pectral **A**bundance **F**actor, mean average of three biological replicates; the NSAF normalizes across samples and takes protein sizes into account; values range between 0 and 1, increasing values indicate higher abundance [27], stdev standard deviation of the means of three biological replicates; probability ranges associated with Students t-test (Scaffold 4.0.5).

3 ND not detected (NSAF 0 in at least two biological replicates and <0.005).

*due to high sequence similarity all peptide hits for “flagellin” (Q81FD3, Q81FD4, Q81FD5) were combined.

Due to frequent flagellar turnover, flagellum structural components are common constituents of bacterial secretomes [28]–[31]. In agreement with the observed motility deficiency of the mutant and the highly reduced number of flagella seen in AFM experiments, levels of several flagellum structural proteins were reduced in the Δ*secDF* secretome. *B. cereus* ATCC 14579 encodes three highly similar flagellin proteins (Q81FD3, Q81FD4, Q81FD5), whose peptide fragments could not be distinguished from each other by the applied analysis method and were therefore analyzed together. In total, slightly less flagellin was detected in the Δ*secDF* mutant growth medium (60% of wild type level, p = 0.049). Furthermore, the cell-wall associated hook protein FlgE was detected in one of three mutant replicates only (4% of wild type level, p-value 0.12, table S3), and the three structural flagellar hook-associated proteins 1, 2 and 3 were present on average 18%, 40% and 52% of the wild type levels, respectively (p-values  = 0.06, 0.35, 0.08, table S3).

Several cell wall-associated proteins were also found to be differentially secreted in the Δ*secDF* mutant, most prominently the putative murein hydrolases BC0991 and BC1991 which were absent in the medium of the Δ*secDF* mutant (Table 1). Furthermore, EntB (BC2952), annotated as enterotoxin/cell-wall binding protein, was present at 33-fold lower levels (p<0.001) and was, in fact, not detectable in two out of three biological replicates. The similar proteins EntA (BC5239) and EntC (BC0813) did not show this trend, as the abundances varied across the samples.

In contrast to the less abundant proteins in the Δ*secDF* secretome, most of the 18 proteins found at higher levels in the growth medium of the mutant relative to wild type typically had intracellular functions, including ten ribosomal proteins, with sizes ranging between 11 and 25 kDa. Finally, it is also worth noticing that the so far uncharacterized putative enterotoxin BC1953 was among the most abundant proteins in the wild type secretome at the time of sampling (Table S3).

### Toxin translocation is reduced in the ΔsecDF mutant

**Figure 4:**
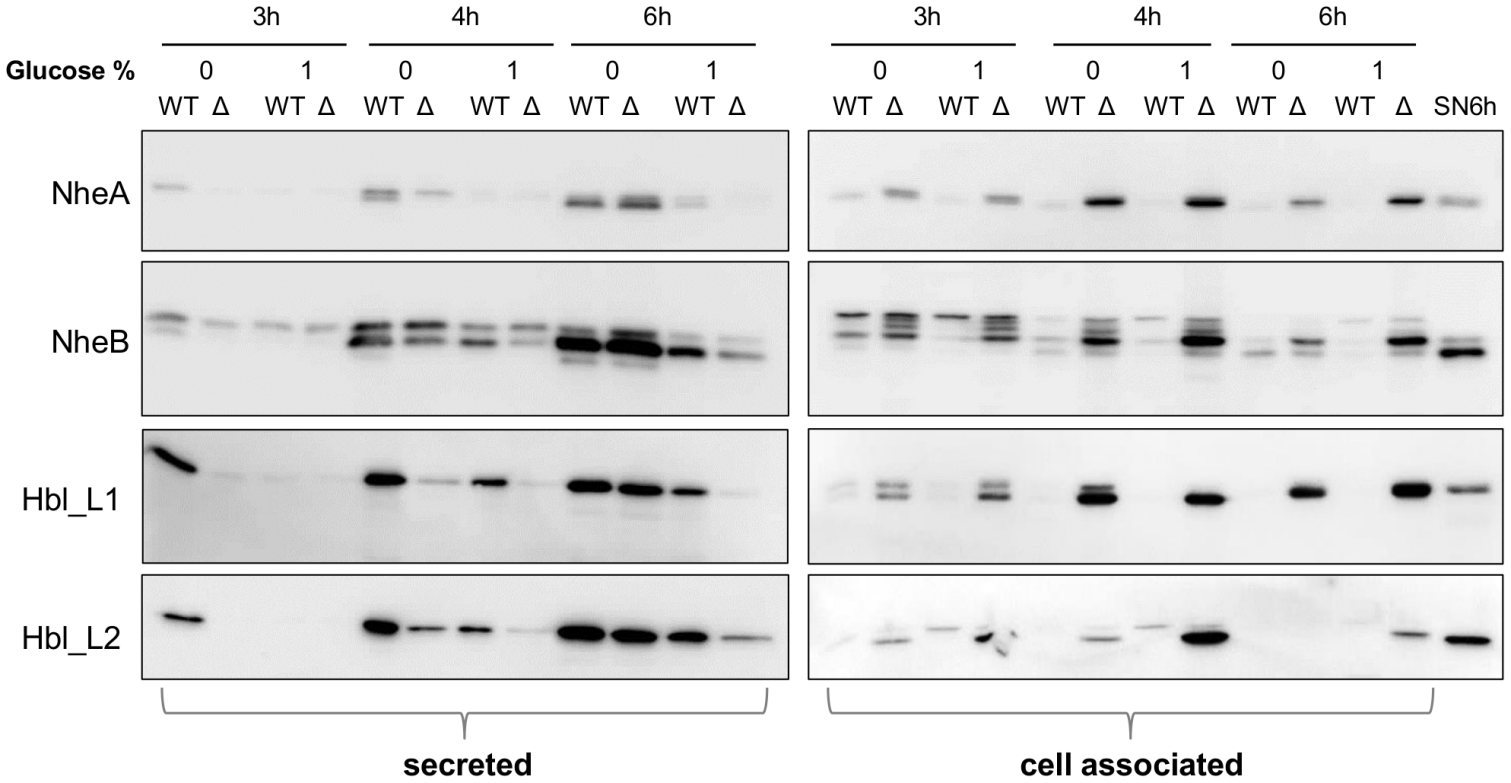
Comparison of NheA, NheB, Hbl_L1 and Hbl_L2 secretion by western-blot analysis. Western-blot assay of secreted (**left**) and cell associated (**right**) toxin components NheA, NheB, Hbl_L1 and Hbl_L2. Samples of the growth medium were taken from the wild type (WT) and the Δ*secDF* mutant (Δ) from 3 h (exponential phase), 4 h (transition phase) and 6 h (stationary phase) cultures with and without added glucose. The blots are representative of at least two biological replicates. To visualize size differences between pre- and mature proteins, a supernatant wild type sample (SN6H) has also been applied to the blot showing cell associated protein.

Mass spectrometry analysis of the Δ*secDF* secretome indicated a potentially important function for the SecDF moiety in translocation of *B. cereus* proteins, including toxins and other virulence factors. To further characterize this phenomenon, Western blot analyzes were conducted on both the growth medium and cell lysates using monoclonal antibodies against the Hbl toxin components L1 and L2 as well as against NheA and NheB [32], [33] (Fig. 4). In the absence of added glucose, the level of toxin components in the growth medium was reduced in the Δ*secDF* mutant compared to the wild type after 3 h, 4 h and 6 h in LBG medium, but reached wild type levels after 6 h incubation in LB medium (Fig. 4). Over the same time period, cell-associated toxin components accumulated to a higher level in the Δ*secDF* mutant compared to the wild type (Fig. 4). Complementation assays in the Δ*secDF* mutant restored its ability to translocate and averted the cellular accumulation of the indicated toxin components (Fig. S3). Thus, differences in protein abundances in the Δ*secDF* mutant secretome are most likely due to inhibition of toxin translocation across the plasma membrane rather than downregulation of toxin gene transcription or translation. In general, extracellular toxin levels decreased both in wild type and Δ*secDF* mutant cells when grown in the presence of added glucose (Fig. 4), however, the difference in cellular accumulation of toxin components in the Δ*secDF* mutant relative to the wild type was most prominent in cultures grown in LBG rather than in LB (Fig. 4).

**Figure 5:**
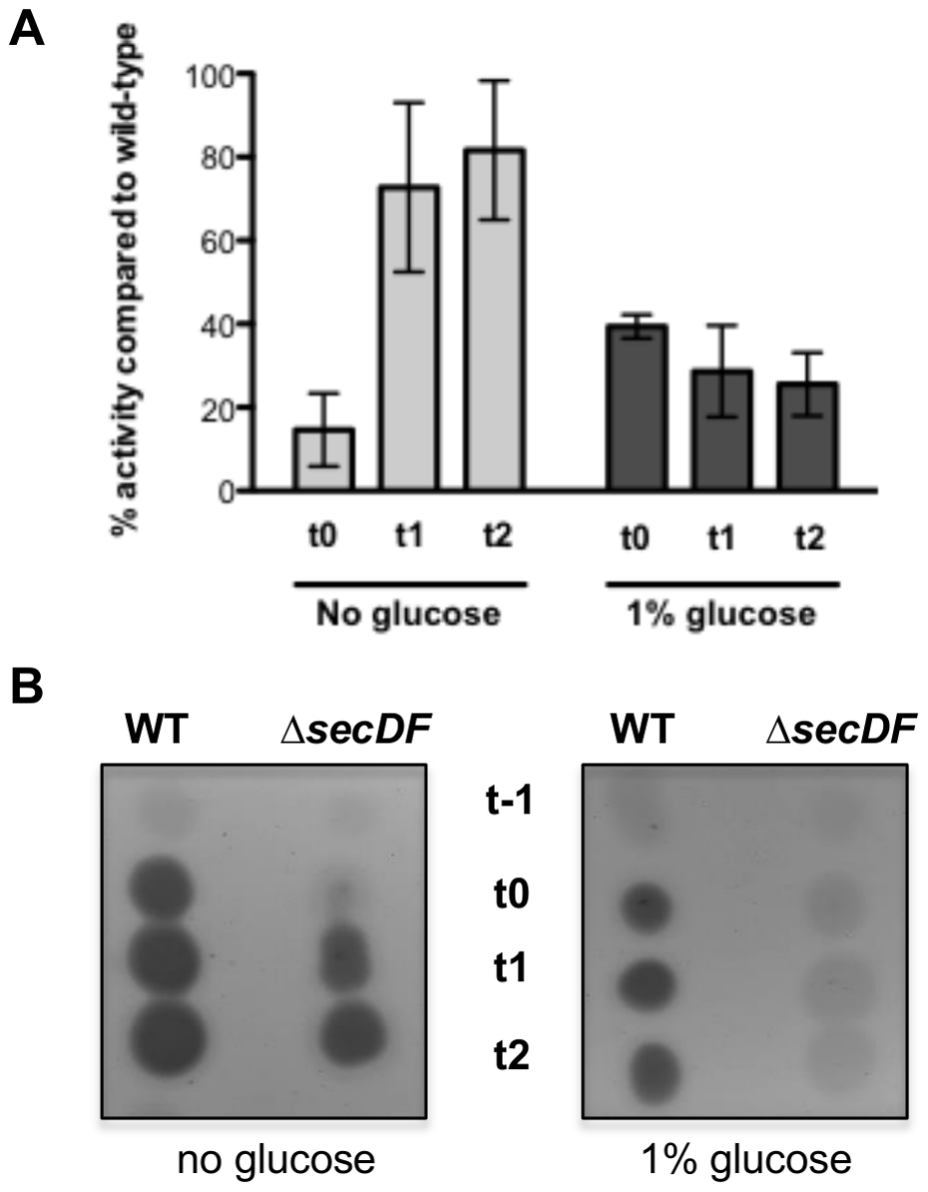
Reduced PC-PLC activity in the Δ*secDF* mutant. A PC-PLC activity assay indicates reduced enzyme activity in the Δ*secDF* mutant compared to the wild type strain. **A**) Filter-sterilized supernatant of cultures grown in LB (no glucose) or LBG (1% glucose) were assayed in a 2% egg-yolk solution. The results are the mean values of two independent experiments, and error bars represent standard deviations. **B**) Five µl of filter-sterilized supernatant of cultures grown in LB or LBG were spotted on 1% egg yolk agar plates. t0 marks the transition point of growth into stationary phase, and t*n* is the number of hours before (-) or after t0. The pictures represent one of two independent experiments.

While PC-PLC was the second most abundant protein in the wild type secretome, levels in the Δ*secDF* mutant culture medium were below the detection limit (Table 1). To confirm the proteome data, both strains were grown in LB and LBG, and culture medium was collected periodically. The PC-PLC activity of sterile-filtered medium on egg yolk agar indicated reduced PC-PLC secretion by the Δ*secDF* mutant (Fig. 5B). Notably, growth in LBG resulted in hardly any visible PC-PLC activity in the mutant culture. Simultaneously, secretion of PC-PLC into the agar by actively growing cells was not detected on LBG agar containing egg yolk (Fig. S2A). In a more sensitive approach to determine PC-PLC activity, culture supernatants were incubated with egg yolk suspension and the substrate degradation measured photometrically (Fig. 5A). These experiments showed that extracellular PC-PLC activity from the *secDF* mutant grown in LBG remains at about 35% of the wild type activity over the studied time course. While the presence of glucose reduced PC-PLC activity in both strains, the effect was more pronounced for the mutant (Fig. 5).

### Deletion of SecDF affects virulence in Galleria mellonella

In order to test if the observed reduction of secreted virulence factors in batch cultures is mirrored by diminished virulence of the Δ*secDF* mutant, *in vivo* infection assays using *Galleria mellonella* (*G. mellonella*) larvae were conducted [34]. Survival of larvae 24 h and 72 h post infection either administered by oral feeding or by injection into the insect blood hemocoel was monitored and the LD_50_s of the wild type strain and of the *ΔsecDF* mutant were evaluated by Probit analysis (Table 2). The confidence limits at the 95% interval of lower (LDL) and upper level (UDL) doses of mutant and wild type strains were not overlapping. Thus, the about 4-fold and 3-fold differences in the dose killing 50% of the exposed larvae at 24 h and 72 h post infection by direct injection into the hemocoel of various doses (2x10^3^ to ≈1x10^5^) of vegetative bacteria, respectively, are significant (p-values ≤0.05). Estimation of virulence at 24 h and 72 h post oral infection revealed about 17-fold and 13-fold LD_50_ difference, respectively. These results clearly indicate a reduced virulence of the Δ*secDF* mutant strain towards the insect model which is more pronounced if larvae are infected orally.

**Table 2:** Role of SecDF in virulence against *Galleria mellonella* insect larvae.

	Oral force feeding^b^				Hemocoel injection			
Hours post infection	24 h		72 h		24 h		72 h	
Strain	WT	Δ*secDF*	WT	Δ*secDF*	WT	Δ*secDF*	WT	Δ*secDF*
c **LD_50_ cfu**	**1.5x10^6^**	**26x10^6^**	**1.6x10^6^**	**21.1x10^6^**	**2.2x10^4^**	**9.9x10^4^**	**1.7x10^4^**	**4.6x10^4^**
dLDL cfu	0.1x10^6^	20.4x10^6^	0.2x10^6^	13.3x10^6^	1.4x10^4^	8.2x10^4^	0.09x10^4^	3.3x10^4^
UDL cfu	3.1x10^6^	31.6x10^6^	3.6x10^6^	28.7x10^6^	4.9x10^4^	11.6x10^4^	2.5x10^4^	5.9x10^4^
**Fold difference LD_50_**	**≈17.3** *		**≈13.2** *		**≈4.5** *		**≈2.7** *	

a Infections with mid log phase (OD_600nm_ = 1) vegetative bacteria cultured in LB medium.

b For oral infection the bacteria are mixed with 3 µg/10 µl of Cry1C toxin. The toxin alone results in ≈10% mortality.

c LC_50_, correspond to the bacterial dose (cfu, colony forming units) per larvae, killing 50% of the treated larvae.

d confidence interval at 95% level.

*(*P*-value ≤0.05). LDL (lower dose limit), UDL (upper dose limit). Mortality was estimated by Probit analysis (StatPlus). based on at least two independent experiments. Control experiments were run with buffer (PBS) and no mortality occurred within 72 hours at 37°C.

### Transcriptional profiling of the ΔsecDF mutant reveals the induction of multiple cellular stress responses

With the purpose of revealing molecular mechanisms linking the protein secretion defect and potential underlying processes to the observed phenotypic changes of the Δ*secDF* mutant, a global transcriptional profiling experiment was conducted. Custom-made microarray slides were hybridized with reverse transcribed RNA extracted from wild type and Δ*secDF* mutant cells at 3 h (two biological replicates) and 4 h (six biological replicates) of cultivation in LBG, on the basis that these time points mark the onset of morphological changes in the mutant compared to wild type. Significant differential expression was observed in more than 400 genes (>2-fold differential expression) during the transition phase (4 h). Table 3 lists 70 genes that exhibited confidently more than 5-fold differences in transcription levels at the 4 h time point. Quantitative RT-PCR confirmed the expression trend for 17 out of 18 selected genes (Fig. S4). In general, genes involved in metabolism and energy conversion processes, membrane transport, resistance and detoxification mechanisms, and motility, as well as several hypothetical genes, were most strongly affected. Furthermore, genes indicative of a cell wall stress response were stimulated in the Δ*secDF* mutant (Table 3), including a phage shock response (*pspA*-like BC1436) gene, and an operon encoding a putative sigma W-type extracytoplasmatic function (ECF) sigma factor (BC5361-BC5363). Transcription of the genes *entA* (BC5239) and *entC* (BC0813) coding for putative cell-wall binding proteins were also upregulated. The *entB* gene (BC2952) showed a lower transcription level as well as a reduced amount of the EntB protein in the extracellular medium of the Δ*secDF* mutant.

**Table 3:** Genes with at least a five-fold differential transcription level in the *ΔsecDF* mutant compared to the isogenic wild type strain *B. cereus* ATCC 14579.

	Locus_tag^1^	Genbank_annotation	FC^2^	P–value^3^
***Resistance/Detoxification***	BC2984	Immune inhibitor A precursor	9.79	2.9E-07
	BC2985	Vancomycin B-type resistance protein vanW	8.63	1.6E-06
***Transport***	BC0816	periplasmic component of efflux system	5.01	2.9E-07
	BC3586	Oligopeptide-binding protein oppA	0.18	2.7E-03
	BC3788	Nucleoside transport system permease protein	0.06	2.9E-07
	BC3790	Nucleoside transport ATP-binding protein	0.11	3.5E-05
	BC3791	Nucleoside-binding protein	0.06	1.7E-06
	BC3792	Transcriptional regulator, GntR family	0.09	1.4E-05
	*BC4405*	*Protein translocase subunit SecDF*	0.13	1.0E-05
	BC4831	ABC transporter ATP-binding protein	6.68	3.9E-08
	BC5117	ABC transporter permease protein	0.11	1.6E-06
	BC5118	ABC transporter ATP-binding protein	0.12	3.1E-05
	BC5253	ABC transporter permease protein	0.08	9.1E-06
	BC5254	ABC transporter ATP-binding protein	0.11	5.0E-06
	BC5255	periplasmic component of efflux system	0.08	8.2E-07
***Metabolism***	BC0297	Guanine-hypoxanthine permease	0.08	9.2E-08
	BC0323§	PRAI carboxylase catalytic subunit	0.04	2.4E-08
	BC0324§	PRAI carboxylase ATPase subunit	0.07	2.1E-08
	BC0325§	Adenylosuccinate lyase	0.07	3.7E-07
	BC0326§	PRAI-succinocarboxamide synthase	0.04	2.4E-05
	BC0327§	PRFGA synthetase, PurS component	0.04	4.6E-06
	BC0328§	PRFGA synthase	0.04	1.9E-06
	BC0329§	PRFGA synthase	0.04	1.6E-06
	BC0330§	Amidophosphoribosyltransferase	0.04	4.8E-06
	BC0331§	PRFGA cyclo-ligase	0.04	6.2E-07
	BC0332§	Phosphoribosylglycinamide formyltransferase	0.05	1.6E-06
	BC0333§	IMP cyclohydrolase	0.06	6.1E-06
	BC0491	Formate acetyltransferase	0.18	2.1E-04
	BC0492	Pyruvate formate-lyase activating enzyme	0.15	7.7E-04
***Respiration***	BC1939	Cytochrome d ubiquinol oxidase subunit II	6.31	2.3E-05
	BC2119	Respiratory nitrate reductase beta chain	0.07	2.2E-04
	BC2120	Respiratory nitrate reductase delta chain	0.20	3.2E-02
	BC4792	Cytochrome d ubiquinol oxidase subunit I	0.14	8.6E-05
	BC4793	Cytochrome d ubiquinol oxidase subunit II	0.11	8.1E-04
***Putative Cell Wall Stress Response***	BC0813	enterotoxin/cell-wall binding protein entC	6.35	6.3E-07
	BC1435	hypothetical protein	33.96	2.1E-08
	BC1436	Phage shock protein A	12.83	7.9E-07
	BC5239	enterotoxin/cell-wall binding protein entA	5.60	7.9E-07
	BC5361	ECF-type sigma factor negative effector	12.40	1.7E-06
	BC5362	ECF-type sigma factor negative effector	8.26	2.4E-08
	BC5363	RNA polymerase ECF-type sigma factor	16.82	4.8E-07
***Motility***	BC1657	Flagellin	0.18	1.8E-06
	BC1659	Flagellin	0.19	6.7E-05
***Sigma B operon***	BC0862	Protease I	15.77	1.3E-05
	BC0863	Catalase	13.31	4.2E-06
	BC0998	General stress protein 17M	11.41	2.1E-08
	BC0999	hypothetical protein	12.27	2.8E-07
	BC1000	hypothetical Membrane Spanning Protein	12.54	6.7E-06
	BC1002	Anti-sigma B factor antagonist	5.36	2.5E-06
	BC1003	Anti-sigma B factor	8.97	1.4E-06
	BC1004	RNA polymerase sigma-B factor	7.84	1.8E-06
	BC1010	hypothetical protein	10.61	4.5E-06
	BC3130	hypothetical protein	5.30	7.4E-05
***Others***	BC0494	hypothetical Cytosolic Protein	0.19	6.7E-06
	BC1760	3-oxoacyl-[acyl-carrier-protein] synthase III	5.06	2.6E-06
	BC1852	Exonuclease SbcC	0.20	3.4E-04
	BC1854	hypothetical Cytosolic Protein	0.20	1.4E-04
	BC1861	DNA/RNA helicase (DEAD/DEAH box family)	0.20	3.2E-05
	BC2056	hypothetical protein	0.16	3.4E-07
	BC4482	hypothetical protein	5.32	6.5E-05
	BC4813	hypothetical protein	14.25	1.8E-07
	BC5116	hypothetical protein	0.16	1.3E-05
	BC5119	hypothetical protein	0.12	2.8E-05
	BC5120	hypothetical Cytosolic Protein	0.12	6.7E-06
	BC5121	hypothetical protein	0.12	1.7E-05
	BC5122	hypothetical Cytosolic Protein	0.18	2.4E-05
	BC5123	hypothetical protein	0.16	3.6E-05
	BC5124	hypothetical protein	0.19	2.7E-05
	BC5243	hypothetical protein	0.20	9.1E-05
	BC5252	hypothetical Membrane Spanning Protein	0.11	2.3E-06

1 data on the linear plasmid pBClin15 can be found in the supplementary file.

2 FC fold change of transcriptional expression in *B. cereus* Δ*secDF* compared to wild type.

3 P-values were computed using false discovery rate correction of 0.05 by an Bayesisn linear model as integrated in the Limma-package [90]; data represent six independent cultures.

§ purine operon under the control of PurA.

At the 4 h time point the Δ*secDF* mutant showed a highly activated sigma B stress response regulon compared to the wild type (Table 3, Table S4). To confirm this, expression of *sigB* was followed over time by real-time quantitative PCR. While there were no significant changes (p<0.5) between the Δ*secDF* mutant and the wild type strain at early and mid-exponential growth phase, *sigB* was 3- to 17-fold induced in the mutant compared to the wild type at late-exponential and transition phase (p<0.01; data not shown). In total 14 out of 26 previously described heat-shock activated, sigma B-dependent genes [35], were more than two-fold upregulated in the Δ*secDF* mutant (Table S4). The most strongly induced genes in the Δ*secDF* mutant were also among the highest heat-shock induced genes (e.g. those encoding KatE and Protease I). In order to test if these transcriptional changes translated into a cellular phenotype, the catalase activity of cultures grown for 6 h was measured. In support of the activation of the SigB operon, the Δ*secDF* mutant exhibited approximately 20% increased catalase activity (data not shown), however only when grown in the presence of glucose.

Secretome analysis of the *B. cereus ΔsecDF* mutant had revealed strongly reduced levels of virulence factors in the supernatant, which was confirmed by Western blot analyses of cell-accumulated and extracellular Hbl and Nhe toxin component levels, thus indicating SecDF-mediated export. Transcriptional levels were also altered for several (*plcB, smase, colC*, BC2552, *nprB*), but not all (*cytK*, *nhe, hbl*) PlcR-regulated virulence determinants (Table S4, S5). PlcR plays a key role in pathogenicity as it acts as a transcriptional regulator of many extracellular virulence factors. *plcR* transcription is autoregulated and the activity of the protein depends on the signaling peptide PapR [36], [37]. However, PlcR was not differentially expressed over the course of 4 h growth (data not shown). It is nevertheless noteworthy, that the oligopeptide permease system, BC1179-BC1183, which is responsible for re-import of the PapR pheromone after extracellular cleavage [38], was transcriptionally downregulated in the Δ*secDF* mutant (Table S5).

Interestingly, almost all motility-associated genes (BC1625-BC1671) were consistently downregulated two-fold or more in the mutant at the 4 h time point. Thus, the observed reduced flagellation and motility of the mutant was possibly due to reduced transcription of motility-associated genes encoding flagellar components and chemotaxis proteins.

Other prominent transcriptional responses due to *secDF* deletion were the stimulation of the cysteine regulon of CymRD, and downregulation of purine metabolism. Furthermore, seven uncharacterized ABC-transporters (out of a total of 111 [39]) were more than 2-fold differentially regulated, as were 98 hypothetical protein-encoding genes (Table 3 and S5). Genes known to be activated by anaerobic conditions at low oxygen pressure or high culture densities [40]–[42] were downregulated in the Δ*secDF* mutant (Table 3 and Table S5). This encompassed factors involved in oxidative phosphorylation (operons BC3941-3944; BC0695-0698) including a cytochrome d ubiquinol oxidase (BC4792-4793), fermentation (BC0491-0492, BC2220), anaerobic respiration (BC2134, BC2128) and the regulator of the arginine deaminase operon arcABDC (BC0410).

The genome of *B. cereus* ATCC 14579 also contains a cryptic, linear plasmid pBClin15, encoding what appears to be a dormant prophage [43]. Most of the pBClin15 genes were found to be downregulated in the Δ*secDF* mutant (Fig. S5). This was not a result of loss of the pBClin15 plasmid, since (i) the presence of ORF 1–3 was detected via PCR using genomic DNA isolated from the bacterial culture used for the microarray analysis, and (ii) mRNA transcripts of BC_p0006 and BC_p0007 were detected by real-time qPCR from an independent culture.

## Discussion

In the present study, deletion of *secDF* in *B. cereus* ATCC 14579 results in a pleiotropic phenotype which includes premature growth arrest and smaller colony size, aberrant cell morphology, reduced motility and reduced total protein in the bacterial secretome, consistent with previous reports on *secDF* mutants in other bacterial species [6], [9], [11], [44], [45]. In addition, our experiments demonstrated more pronounced pleiotropic effects in the presence of glucose.

Nhe, Hbl and Cytotoxin K are well-studied toxins from *B. cereus*, causing the diarrheal syndrome after ingestion of contaminated food [2]–[4]. The *nhe* and *hbl* operons in the wild type and mutant strains were not found to be differentially transcribed, while Western blotting experiments using monoclonal antibodies showed accumulation of Hbl and Nhe toxin components in the Δ*secDF* mutant cells (Fig. 4, Fig. S3). A Sec-translocase - mediated export of these toxins has been advocated by Fagerlund *et al.* [5]. However, it has also been indicated that the Hbl enterotoxin as well as the PC-PLC may be secreted via the flagellar apparatus [46], [47], similar to what is known in *C. jejuni* [48] and *C. difficile* [49]. Since the transcription of the flagellar machinery is downregulated in the Δ*secDF* mutant we cannot state explicitly whether the translocation defect of Hbl components is due to secondary effects on the flagellar system or to direct inhibition of the Sec-translocase pathway. For virulence factors other than Hbl and Nhe, such as cytotoxin K, PLC, SMase and collagenase C, a weak to moderate, yet statistically significant, transcriptional downregulation was observed. PlcR is a key transcriptional regulator involved in integration of a range of environmental signals such as cell-density and nutrient deprivation, and controls the expression of a range of extracellular *B. cereus* virulence factors, including Nhe, Hbl, CytK, PC-PLC and SMase. Interestingly, CytK, PLC, SMase, BC0991 and ColC were among the highest differentially detected proteins in the culture supernatants.

Knowing that the Δ*secDF* mutant has such a strong impact on secretion of known virulence factors and that the respective *S. aureus* and *L. monocytogenes* SecDF null mutants were affected in virulence [12], [50], we sought to evaluate the role of *B. cereus* SecDF in its capacity to kill the insect larvae *Galleria mellonella*, which is currently used for infection studies of *B. cereus* or *B. thuringiensis* strains [34], [51]–[53]. Virulence tests were performed by two routes of infection and the strongest effect was recorded following oral infection with about 17-fold more Δ*secDF* bacteria needed to kill 50% of the larvae at 24 hs compared to the wild type (Table 2). In addition, the mutant strain was also 4.5-fold less virulent 24 h post infection when the bacteria were injected into the hemocoel. This indicates that the Δ*secDF* mutant is definitely affected in virulence but it is difficult to appoint the effect to a particular gene set because of the pleiotrophic effect of the mutation. Notably, the differences in virulence decreased after 74 h in both infection model experiments. This indicates that the reduced virulence of the Δ*secDF* mutant might only be of transient nature, a notion supported by Western Blot experiments showing Nhe and Hbl components adapting comparable extracellular levels in both strains over time. Meanwhile the results are in line with former work on the non-motile mutant *B. thuringiensis* 407 cry^−^ Δ*flhA*, where a defective flagellar machinery assembly led to a decrease in virulence [51]. This was found to be partly due to a reduction of virulence gene expression, rather than direct involvement of the flagellar apparatus in virulence factor secretion [5], [51]. Since flagellar gene expression is reduced in the Δ*secDF* mutant, the extent to which Hbl is transported via the Sec-translocase and the flagellar mechanism, respectively remains to be determined.

Out of the 96 proteins that could be identified in the *B. cereus* ATCC 14579 and isogenic Δ*secDF* mutant secretomes (Table S3), the majority (57%) were predicted to be of cytoplasmic origin. Other studies also frequently report a high percentage of non-secretory proteins in the medium [31], [54], [55], and cell lysis has been determined to be of only minor contribution [56]–[58]. In LBG medium the Δ*secDF* mutant did not exhibit increased autolysis compared to the wild type (data not shown). Cytoplasmic proteins like enolase and pyruvate dehydrogenase were detected in the growth medium of *B. cereus* (Table S3 and [29]), and these and other intracellular proteins have been reported to be secreted in *B. subtilis* during stationary phase by a non-classical translocation mechanism where protein domain structure appears to contribute [59]. Although we did not find any indication of a stronger autolysis in the Δ*secDF* mutant compared to the wild type, an increased amount of small sized ribosomal proteins was identified in the growth medium of the mutant (Table 1). No difference was seen at the transcriptional level of these genes between the wild type and the mutant. During co-translational insertion of proteins into the cell membrane the translocation channel protein SecY is bound to the ribosomal machinery [60], [61] and in fact, it has been shown recently that this interaction opens the internal plug of SecY [62]. Based on current knowledge, we cannot rule out the possibility that loss of SecDF could potentially result in a less specific translocation mechanism through a leaky SecYEG complex, feasibly affecting translocation of small sized proteins.

The Δ*secDF* mutant presented an aberrant cell morphology combined with an earlier growth arrest during cultivation (Fig. 1), phenotypes potentially caused by atypical activity of peptidoglycan remodeling enzymes. Murein hydrolases function during cell wall growth, peptidoglycan turnover, cell separation, and autolysis [63]. Two uncharacterized putative murein hydrolases (BC0991 and BC1991) were absent in the Δ*secDF* mutant growth medium (Table 1). Both contain a transglutaminase domain, known to facilitate intra- and interprotein crosslinks and to potentially play an important role in cell wall maturation [64]. In addition the putative cell wall binding proteins EntA (BC5239), EntB (BC2952), and EntC (BC0813), identified in the secretome of *B. cereus* [28], were affected at the transcriptional level (Table S5) and, in the case of EntB, also in the extracellular proteome in the *secDF* deletion mutant (Table S3). Secretome analyses for EntA and EntC were, however, not conclusive. EntA, EntB and EntC all contain two copies of the cell wall-binding SH3 domain, and are members of the resuscitation-promoting factor/stationary-phase survival (Rpf/Sps)-family identified in actinobacteria and firmicutes [65]. The *B. subtilis* muralytic enzyme YocH, which is a homolog of EntA, EntB and EntC, was induced by cell wall-turnover peptidoglycan fragments of growing cells and a null mutant displayed reduced survival after post-exponential phase [66]. Crucial residues for enzyme activity in YocH [66] are conserved in the three putative cell wall-binding proteins EntA, EntB and EntC. Clearly further analysis is required to understand the regulation and involvement of these and other muralytic enzymes in the phenotypic changes of the Δ*secDF* mutant (Fig 1).

AFM images clearly showed a reduction in cellular flagellation in the Δ*secDF* mutant (Fig. 2C), probably as a result of transcriptional deactivation of genes coding for flagella components (Table 3). While intramembranous constituents of the flagellar body are generally believed to be inserted in a Sec-translocase dependent manner, the outer components are secreted via a flagellum-specific type III secretion system [67]–[69]. It is known from *E. coli* and *S. enterica* that the expression of flagellar genes is dependent on the state of assembly, in a step-wise manner (see reviews [70], [71]). Assuming a similar, energy-saving feedback loop in *Bacillus*, it is possible that the transcriptional downregulation of flagellar genes results from incomplete insertion and assembly of intramembrane flagellum body proteins. Thus, one could hypothesize that SecDF plays a role in early flagellum construction in *B. cereus* grown in the presence of glucose (Fig. 2).

A global transcriptional profiling experiment revealed profound transcriptional changes in the Δ*secDF* mutant, a phenomenon seen previously for selected genes in a *S. aureus secDF* mutant [11]. Among the genes most highly upregulated by *secDF* deletion were a range of genes thought to respond to disturbances in cell envelope structures: the phage shock response system, the sigma B regulon, an extracytoplasmatic function (ECF) sigma factor and the putative murein hydrolase BC1991 (Table 3). The PspA-like gene (BC1436) is similar to *liaH* of *B. subtilis*. The Lia operon (LiaIHFSR) is highly conserved in Firmicutes, and the system is a cell envelope stress response activated by peptide antibiotics [72], [73]. PspA is particularly well studied in *E. coli* and is induced by a wide range of cell envelope stress conditions and thought to maintain the energetic state of cells under stress (for review see [74]). In *E. coli* it has been shown that single gene deletions of Sec-translocase components such as SecA, SecD and SecF, lead to PspA overexpression [75], and that PspA supports the efficient translocation of Sec- and TAT-dependent proteins [76]. In our study of the *B. cereus* Δ*secDF* mutant, the strong induction of the *pspA*-like gene may be a result of sensing the secretion defect as well as of an internal accumulation of proteins. In addition, the sigma B regulon known to provide a non-specific stress response to a range of different stress signals affecting cell envelope integrity [77]–[79] is moderately upregulated. Among the ten ECF-type sigma factors identified in *B. cereus* [80], recognizing environmental signals [81], the so far uncharacterized BC5363 exhibits similarity to the *B. subtilis* SigW sigma factor (34% identity at the protein level). Interestingly, *sigW* is induced by cell envelope stress factors (for review see [82]).

While the addition of glucose to the growth medium resulted in general in more pronounced phenotypes (Fig. 1, Fig. 4, Fig. 5), it is noteworthy that only sugars consisting of at least one glucose component profoundly inhibited motility of the *ΔsecDF* mutant (Fig. 2A). Although the rationale and mechanism behind the effects of glucose on the phenotype of the Δ*secDF* mutant remain to be elucidated, this study confirms previous reports showing that glucose exerts more functions than only being an important nutrient. Recent research indicates for instance a direct involvement of glucose in expression of the toxin hemolysin II in *B. cereus* by activation of HlyIIR by glucose 6P which resulted in repression of *hlyII* gene expression [83].

The present study shows that some toxins and other virulence factors produced by the pathogenic Gram-positive, spore-forming bacterium *B. cereus* are dependent on SecDF for proper translocation across the cell membrane, confirming a role for SecDF in protein secretion in general and efflux of some toxins, directly or indirectly, in particular. It could be assumed that the ubiquitous SecDF protein fills similar functions also in other bacteria, as it has been reported for *S. aureus* and *L. monocytogenes* [12], [50]. Finally, although we cannot explain the phenomenon at this moment, this study shows clearly an exacerbating effect of glucose on the phenotype of the Δ*secDF* mutant.

## Materials and methods

### Growth conditions

Unless otherwise stated, *B. cereus* and *E. coli* strains were streaked on LB agar plates and incubated at 30°C and 37°C, respectively. Liquid cultures were inoculated from a single colony, incubated overnight and then diluted 1∶100 in LB medium. These starter cultures were grown at 30°C or 37°C, respectively, at 200 rpm. After reaching an OD_600nm_ of approximately 0.5, experimental cultures were inoculated from the starter culture to an initial OD_600nm_ of 0.02, and grown as above. If applicable, 1% glucose was added to LB (LBG). When relevant, erythromycin 5 µg/ml (with pHT304 plasmid) or ampicillin 100 µg/ml (with pTTQ18 plasmid) was added to the culture. For assessment of glucose fermentation the strains were streaked on *Bacillus cereus* agar (Oxoid) supplemented with 1% glucose. Acidic by-products of glucose fermentation were monitored by color change of the pH indicator bromothymol blue.

### Construction of the Δ*secDF*-mutant

The markerless Δ*secDF* mutant of the type strain *B. cereus* ATCC 14579 was constructed by the method of Janes & Stibitz [84]. A deletion construct consisting of overlapping flanking regions of the target gene is cloned into a temperature-sensitive shuttle vector carrying the homing endonuclease restriction site *I-SceI*. Under replication non-permissive temperatures and selection pressure the vector integrates either up- or downstream of the target gene. To enforce a double-strand break of the chromosomal DNA, a second plasmid encoding I-SceI is introduced into the organism. Repair of the break by cross-over leads to either wild type or knock-out genotypes. Mutants are then selected by PCR and the vector sporadically lost during non-selection. Oligonucleotides used for making the gene deletion construct, substituted the BC4405 ORF in frame with ATGGTCGACTAA and thus introduced a *SalI* restriction site (supplemental information S1). After cloning of the gene deletion construct with about 500 bp flanking regions into the suicide shuttle vector pBKJ236 and electroporation into *B. cereus* ATCC 14579, the protocol was followed as previously described [84]. Successful gene deletion was confirmed by PCR using genomic DNA as template and oligonucleotides binding outside of the deleted region, and by DNA sequencing. The presence of the plasmid pBClin15 was confirmed by PCR as reported previously [85].

### Assessment of phospholipase C activity

The activity of secreted Phospholipase C (PC-PLC) was measured for cells growing on agar and in liquid cultures. For the first test, bacteria were grown in LB medium for 16 h at 30°C and 220 rpm, washed in 0.9% NaCl and resuspended to an OD_600nm_ of 8.5. Five µl of the bacterial suspension was spotted onto LB and LBG agar plates supplemented with 5% egg yolk suspension (Oxoid). The phospholipase C activity was analyzed by visual inspection after 7 hours incubation at 30°C. PC-PLC activity of filter-sterilized supernatant sampled at different time points, from cultures grown in LB and LBG, was measured by spotting 5 µl on 1% egg yolk agar plates, and incubating them at 30°C for 24 h. In addition, 100 µl of these supernatants were incubated with 900 µl 2% egg yolk saline suspension at room temperature for 75 min after which the OD_600nm_ was measured. Variations in growth between the wild type and the mutant strains were accounted for when necessary by diluting the wild type supernatant with fresh LB after filter-sterilization.

### Light microscopy and atomic force microscopy (AFM)

Micrographs were made using 3 µl sample of a fresh culture with 400-fold magnification. Pictures were obtained with a Nikon Labophot-2 microscope coupled to a Leica DFC320 camera and assessed with the LAS v3.6 program. For AFM, *B. cereus* ATCC 14579 wild type and Δ*secDF* mutant strains were grown in LBG as detailed under “growth conditions” and one ml samples were collected after 4 h growth. Following 3 min centrifugation at 2400xg the cells were washed and resuspended in 1 ml 0.9% saline. Ten µl of the suspension was diluted to a final volume of 50 µl in 10 mM magnesium/Tris buffer, pH 7.5, ten µl of which was applied to a freshly cleaved muscovite mica (Agar Scientific, Norway) mounted on a glass slide, and incubated for 10 min at room temperature. After ten washing steps with 100 µl sterile filtered MQ water, the samples were dried under a gentle N_2_ stream. AFM images were recorded in intermittent contact mode in air using a NanoWizard I atomic force microscope (JPK, Berlin, Germany). To quantify the number of flagella, a total of 103 cells for the Δ*secDF* mutant and 26 cells of the wild type were analyzed, from two independent cultures.

### Motility assays

To assess motility, 0.3% and 0.7% LB agar plates were used. Five µl of overnight cultures (OD_600nm_ between 7 and 10) of the wild type and mutant strains grown in 5 ml LB at 30°C at 220 rpm were spotted on the agar surface of the same plate, with two technical replicates per biological sample. The diameter of the culture was measured after 7–9 h incubation, the start diameter of the drop was subtracted and the ratio of the recorded motility for wild type and mutant was calculated. Every experiment was done at least four times, and the motility of the wild type strain in each condition was set to 100% (unpaired, two-tailed Student's t-Test for wild type vs. mutant, *P*<0.05). Statistical significance of differences between the mutant's motility compared to the wild type in pure LB and LB + additives was evaluated using the MS Office Excel unpaired t-test function with a two-tailed distribution. Additives were supplemented with the following final concentrations: glucose 0.4%, other sugars 1%, Tween-80; 0.02%.

### Expression of SecDF

For expression of SecDF in *B. cereus* ATCC 14579, the native gene was cloned into the low-copy number *E. coli*/*Bacillus* plasmid shuttle vector pHT304-Pxyl [86]. pHT304-Pxyl contains the *xylR* and *xylA* promoters from *B. subtilis*, allowing xylose-inducible expression of SecDF fused with a C-terminal 6x histidine tag. For heterologous overexpression of SecDF in *E. coli*, the *secDF* gene from *B. cereus* ATCC 14579 was cloned into a modified version of the high copy number, IPTG inducible vector pTTQ18 [87]. Expression of *secDF* from this plasmid resulted in a recombinant protein carrying a C-terminal 6x histidine tag. The plasmid was introduced into *E. coli* BW25112 Δ*acrB*. This strain lacks the RND-type transporter AcrB, which has been shown to be the major xenobiotic efflux transporter in *E. coli* (for recent reviews see [88], [89]). Correct cloning of the gene was in both cases confirmed by sequencing, and protein expression in both host organisms was measured using the histidine tags for detection by specific antibodies. Induction of protein expression by 20 mM xylose and 0.05 mM IPTG, respectively, resulted in a protein band of approximately 82 kDa on a Western blot, in both cases (data not shown).

### Determination of minimum inhibitory concentrations (MICs)

To identify the susceptibility of *B. cereus* and *E. coli ΔacrB* to a range of xenobiotics, bacterial suspensions were incubated in LB and LBG, respectively, with 2-fold serial dilutions of the tested compounds. Pre-cultures grown in LB were diluted to an OD_600nm_ value of 0.02 and aliquoted into 96-well plates (final volume 150 µl). The plates were incubated in a humidified chamber at 30°C, 200 rpm for 22 h. The lowest concentration of xenobiotics that resulted in no visual growth was considered as the MIC. Experiments were done in technical duplicates and with at least two biological replicates. If protein overexpression strains were tested, xylose (20 mM) or IPTG (0.05 mM) was added to the medium for pHT304-Pxyl and pTTQ18 vector constructs, respectively. Alternatively, the susceptibility of *B. cereus* strains was examined by disk diffusion on LB or LBG agar plates. Mid-logarithmic precultures were diluted to an OD_600nm_ of 0.05 in 0.9% NaCl, and 1 ml of this cell suspension was spread out on agar plates and air-dried. Thereafter, 6 mm paper disks applied on the surface were impregnated with 10 µl of each tested compound. Inhibition zones were examined after 16 h incubation at 30°C, for the following compounds: ethanol 100%, spectinomycin 100 mg/ml, phosphomycin 25 mg/ml, ciprofloxacin 10 mg/ml, norfloxacin 10 mg/ml, chloramphenicol 25 mg/ml, tetracycline 10 mg/ml, oxytetracycline 0.8 mg/ml, gentamicin 50 mg/ml, ampicillin 50 mg/ml, oxacillin-5 (BD), SDS 20%, DOC 80 mg/ml, chlorhexidin 1.6 mg/ml, ethidium bromide 5 mg/ml, CCCP 7.5 mM, sodium lactate 50%, polymyxin B 25 mg/ml, sodium benzoate 0.5 g/ml, erythromycin 100 mg/ml, kanamycin 10 mg/ml, plant extracts: tea tree (*Melaleuca alternifolia*); steam distillates of peppermint leaves (*Mentha piperita*) and calabash (*Melaleuca leucadendron var. cajaputi*) (Primavera Life).

### Microarray analysis

Cells were grown in LBG in 50 ml cultures in 500 ml non-baffled Erlenmeyer flasks at 30°C, 220 rpm for 3 h (two biological replicates) and 4 h (six biological replicates), respectively. Five ml culture was then mixed with equal amounts of ice-cold methanol, followed by harvesting by a short centrifugation. Cells were lysed by beadbeating and the RNA was isolated using the RNA Mini Kit (Qiagen), including the on-column DNase treatment step. cDNA conversion and labelling, microarray hybridization and data analysis using Bayesian linear modelling (Limma-package [90] was basically performed as described previously by Gohar *et al.* [91] and detailed procedures and raw data were deposited according to MIAME guidelines in the Arrayexpress database (accession number E-MTAB-1759).

### Validation of gene expression by real-time RT-PCR analysis

Quantitative real-time PCR (qRT-PCR) was used to validate the microarray results [92], [93]. qRT-PCR was carried out following the MIQE guidelines (supplemental information S1). The genes tested included non-differentially (FC<1.5: *BC_p006, BC2271, ccpA, plcR, hlyR, nheB*), moderately (1.5<FC<5: *BC1991, BC5239, Flagellin, cytK, hlbB, hlyII*) and highly differentially (FC>5: *BC_p007, BC0862, BC1436, BC2119, ECF-type sigma factor, sigB*) expressed genes from the microarray experiment, in order to best mirror the expression pattern observed in the microarray experiments.

### Analysis of secreted proteins

For the analysis of secreted proteins, mid-logarithmic cultures of the *B. cereus* ATCC 14579 wild type and the isogenic Δ*secDF* mutant strains grown in LB were transferred into fresh LB or LBG medium. Following 3 h, 4 h and 6 h aerated growth at 30°C and 220 rpm, PBS-adjusted volumes (by dilution according to 1 ml culture with lowest OD_600nm_) of each culture were harvested by centrifugation. Sterile-filtered (0.2 µm) culture supernatant was mixed 1∶4 with ice-cold methanol:acetone (1∶1) and proteins were precipitated overnight at −20°C. Proteins were harvested by centrifugation at 12,000xg for 30 min at 4°C. For gel electrophoresis, 8 ml of duplicate, independent and normalized supernatants of cultures grown in LBG were concentrated 40-fold by methanol:acetone precipitation, resuspended in 250 µl TES (20 mM Tris pH 7.5, 0.8% NaCl, 1 mM EDTA), and 12 µl of each sample was analyzed on a 4–20% SDS-polyacrylamide gel (Pierce) by silver staining (Sigma-Aldrich). For label-free mass spectrometry analyses, triplicate independent cultures (from individual colonies) were grown for 4 h in LB added 1% glucose, as described above. Using acid-cleaned glassware, the PBS-adjusted culture supernatants (according to the culture with lowest OD_600nm_; final volume of 2.5 ml) were subjected to methanol:acetone precipitation at −20°C overnight. After centrifugation at 12.000xg for 30 min at 4°C, proteins were resuspended in 50 mM ammoniumbicarbonate/1 M urea. After protein concentration determination using the Bradford Assay with BSA as a standard, 20 µg of each sample was used for analyses.

#### Sample Preparation

Proteins were reduced with 10 mM DTT (1 h at 70°C, pH 9), alkylated for 1 h using 25 mM iodoacetamide, and digested with trypsin (1 µg) at 37°C for 16 h. Digested protein samples were analysed using a TripleTOF 5600 mass spectrometer (AB SCIEX Foster City, CA, USA) coupled to an Eksigent NanoLC-Ultra 2Dplus system (Eksigent Technologies, Dublin, CA, USA). Peptides were separated as described previously [94], and the LC eluent subjected to positive ion nanoflow analysis using an ion spray voltage, heater interface temperature, curtain gas flow and nebulizing gas flow of 2.5 kV, 150°C, 20°C and 16°C, respectively. Information dependent acquisition-experiments utilized a survey scan (350–1500 amu) with an accumulation time of 100 ms, followed by 15 MS/MS product ion scans (350–1600 amu) with an accumulation time of 100 ms each.

#### Protein Identification

Proteins were identified using the Paragon search algorithm [95], [96] in ProteinPilot Version 4.0.8085 (AB SCIEX Foster City, CA, USA). Searches were carried out against the reference proteome of *B. cereus* ATCC 14579, extracted from the Universal Protein Resource (UniProt) (4) using the thorough search mode and included biological modifications, trypsin-cleaved peptides and iodoacetamide-modification of cysteine residues. False discovery rates were determined in ProteinPilot using a detected protein threshold of 0.05 and the decoy database searching strategy, and only proteins at 1% global FDR and distinct peptides at 5% local FDR were reported. For further data analysis of all three biological replicates, Scaffold (version Scaffold_4.0.5, Proteome Software Inc., Portland, OR) was used to validate MS/MS based peptide and protein identifications. Equal amount of total protein was used for tryptic digestion and comparative analyses were conducted after normalization of the data sets accordingly to the **N**ormalized **S**pectral **A**bundance **F**actor (NSAF) approach using total spectral counts [27]. A Student's T-test comparing total spectral counts was performed to determine statistical significances of protein abundances in wild type and mutant strain samples.

### Toxin detection

Cell lysates were prepared by harvesting 2 ml of *B. cereus* wild type and Δ*secDF* mutant cultures by centrifugation at 4500xg for 5 min. The pellets were washed once in cold PBS and stored over night at −20°C. Cell pellets were then resuspended in TES containing 2 mg/ml lysozyme and the volume was adjusted according to the original culture OD. The bacterial suspensions were incubated at 37°C for 1 h. After partial cell wall degradation, cell lysis was achieved by six rounds of freezing and thawing in liquid nitrogen and a 37°C water bath respectively. Cell debris was removed by centrifugation and the supernatant was stored on ice for no more than 4 h. Twenty µl of normalized, sterile-filtered supernatants and 2 µl of cell lysates were separated on 10% SDS polyacrylamide gels and blotted onto a nitrocellulose membrane. Toxin components were detected using 1∶20 dilutions of the following monoclonal antibodies: 1A8 and 1E11, against NheA and NheB, respectively [33]; and 1E9 and 8B12, specific for the L1 and L2-subunits of Hbl [32]. 1∶10,000 dilution of HRP-conjugated anti-mouse antibody (Sigma) was used for chemiluminescent signal development.

### Analyses of proteolytic activity

Experiments analyzing milk and gelatin proteolytic activities in the secretomes of the wild type and Δ*secDF* mutant strains did not reveal significant differences (data not shown). Skim milk agar plates were prepared by dissolving skim milk powder and agar separately in Milli-Q water, to a concentration of 75 mg/ml and 15 mg/ml, respectively. Following autoclaving for 15 min at 110°C and cooling to 50°C, the skim milk and agar solutions were mixed (1∶1). Overnight cultures grown in LB broth were normalized to an OD_600nm_ of 1 with 0.9% NaCl and 50 µl was added into punched holes (5 mm) in skim milk plates and incubated at 37°C, 30°C and 20°C, respectively. Proteolytic activity was visible as change in opacity of the milk around the bacterial spots. Total gelatinase activity was carried out as described in Millipores technical publication on gelatin zymography (http://www.millipore.com/userguides/tech1/mcproto009). Ten µl of sterile-filtered and normalized culture of *B. cereus* strains grown for 4 h in LBG at 220 rpm were loaded on an 8% acrylamide gel co-polymerized with 0.1% gelatin, using non-reductive SDS sample buffer. Gelatinase activity appeared as clear bands in the turbid gel background.

### Autolysis

In order to determine if the Δ*secDF* mutant displayed a higher autolysis rate than the wild type strain, two different tests were conducted: (**i**) cell lysis activity of *B. cereus* cell lysates was investigated by performing zymograms using whole *B. cereus* cells as substrate, according to the method of Raddadi *et. al.* [97]; (**ii**) spontaneous autolysis was determined as described by Quiblier *et al.* [11]. Cells grown in LB or LBG, were harvested 4 h after inoculation, washed in 0.9% NaCl, and resuspended in 0.01 M Na-phosphate buffer, pH 7.4 to a final OD_600nm_ of 1. The resulting bacterial suspensions were incubated at 30°C, 200 rpm for 90 min and the decrease in optical density (600 nm) was measured at regular intervals. Neither of the experiments supported a higher autolysis rate in the *secDF* deletion strain compared to wild type when grown in LBG (data not shown).

### Catalase test

The catalase test, based on a stable yellow complex-formation of hydrogen peroxide with molybdate, was carried out basically as described by Góth 1991 [98]. Briefly, *B. cereus* strains were grown in LB or LBG medium at 30°C and 220 rpm. After 3 h and 4 h growth, OD_600nm_ was measured in duplicate, and a volume corresponding to an optical density of 14 per ml was pelleted and resuspended in 100 µl of 6 mM phosphate buffer, pH 7.4. Samples were mixed with 500 µl preheated substrate solution (65 µM H_2_O_2_ in 6 mM phosphate buffer) and incubated at 37°C for 120 sec. The reaction was stopped by adding 500 µl of 32.4 mM ammonium molybdate in 6 mM phosphate buffer. After pelleting the cells, the color change was measured spectrophotometrically in a microplate reader at 405 nm. Each sample was analyzed in triplicate. Absorbance values were subtracted by values of the blank non-reactive wells containing 100 µl of 6 mM phosphate buffer and no bacterial cells. As a loading control, pelleted cells were lysed as described above, and equal volumes of cell lysates were applied on a 12% SDS-polyacrylamide gel. Proteins were stained with Bradford reagent.

### Insect infection experiments

The virulence-related properties of Δ*secDF* were assessed by comparing the killing effect of the *B. cereus* wild type and the Δ*secDF* mutant strains by both oral infection and direct injection into the hemocoel of 5^th^ instar *Galleria mellonella* larvae [34], [99]. *G. mellonella* eggs were hatched at 25°C and the larvae reared on beeswax and pollen. In each experiment, groups of 20 to 30 *G. mellonella* larvae, weighing about 200 mg, were used. For oral infection, the larvae were force-fed with 10 µl of a mixture containing various doses (1.5x10^5^ to 2.5x10^7^) of vegetative bacteria (exponential growth OD≈1in LB medium) and 3 µg of activated Cry1C toxin, prepared as previously described [100]. For injection experiments, the larvae were also infected with vegetative bacteria at various doses, from ≈2,000 to ≈100,000 cfu (colony forming units). Experiments were repeated at least twice. Infected larvae were kept at 37°C and mortality was recorded at 24 h and 72 h post infection. The larvae in the control group were fed PBS buffer. The 50% lethal doses (LD_50s_) values, as estimated using the Probit analysis StatPlus program, corresponds to the cfu killing 50% of the treated larvae.

## Supporting Information

Figure S1 **Susceptibility of the** ***ΔsecDF*** **mutant towards selected compounds.**
(PDF)

Figure S2 **Determination of lecithinase activity.**
(PDF)

Figure S3 **Complementation of the** ***ΔsecDF*** **mutant.**
(PDF)

Figure S4 **Validation of microarray results by qRT-PCR.**
(PDF)

Figure S5 **Regulation of pBClin15 ORFs.**
(PDF)

Table S1 Susceptibility to toxic compounds of *B. cereus* ATCC 14579 wild type strain and its isogenic *ΔsecDF* variant.
(PDF)

Table S2 Susceptibility to toxic compounds of *E. coli* BW25113_ΔacrB expressing SecDF.
(PDF)

Table S3 Secretome of *B. cereus* ATCC 14579 wild type and *ΔsecDF* mutant.
(PDF)

Table S4 Transcriptional activation of the SigB regulon in the *B. cereus* ATCC 14579 *ΔsecDF* mutant compared to its wild type strain.
(PDF)

Table S5 Complete list of microarray results. The list shows at least 2-fold differentially regulated genes in the *B. cereus* ATCC 1459 *ΔsecDF* mutant compared to wild type (P-value <0.05).
(PDF)

Supplemental information S1 **Materials and methods.**
(PDF)
