# Correction: SecDF as Part of the Sec-Translocase Facilitates Efficient Secretion of *Bacillus cereus* Toxins and Cell Wall-Associated Proteins

**DOI:** 10.1371/journal.pone.0111160

**Published:** 2014-10-13

**Authors:** The PLOS ONE Staff

The P-value in the Table 1 heading is incorrect. Please see the corrected Table 1 here.

**Table 1:** Proteins found in different amounts in the culture supernatants of the wild type and ΔsecDF mutant (P-value <0.05).

						WT		Δ*secDF*	
#	Identified Proteins	Localization^1^	locus tag	Uniprot Acc.Nr.	MW (kDa)	NSAF^2^ avg	stdev	NSAF avg	stdev
	**less abundant proteins in the mutant**								
1	Cytotoxin K	EC	BC_1110	Q81GS6	37	**0.14**	0.01	**ND** 3	
2	Enterotoxin / cell-wall binding protein EntB	EC [28]	BC_2952	Q81C32	55	**0.028**	0.004	**0.001**	0.001
3	Perfringolysin O	EC	BC_5101	Q815P0	57	**0.004**	0.001	**ND**	
4	Phospholipase C	EC	BC_0670	Q81HW1	32	**0.55**	0.12	**ND**	
5	Non-hemolytic enterotoxin NheB	EC	BC_1810	Q81EZ7	43	**0.21**	0.05	**0.01**	0.01
6	Putative murein endopeptidase	U	BC_1991	Q81EI5	44	**0.028**	0.007	**ND**	
7	Hemolysin BL lytic component L1	EC	BC_3103	Q7BYC6	44	**0.18**	0.05	**0.01**	0.01
8	Hemolysin BL lytic component L2	EC	BC_3104	Q81BP7	49	**0.25**	0.08	**0.004**	0.003
9	Sphingomyelin phosphodiesterase	EC	BC_0671	Q81HW0	37	**0.28**	0.11	**ND**	
10	putative murein endopeptidase	CW	BC_0991	Q81H34	65	**0.004**	0.001	**ND**	
11	Cell wall endopeptidase, family M23/M37	EC	BC_0740	Q81HR4	42	**0.016**	0.006	**0.001**	0.002
12	Hemolysin BL binding component	EC	BC_3102	Q81BP9	42	**0.10**	0.05	**ND**	
13	Microbial collagenase	EC	BC_0556	Q81I63	109	**0.088**	0.039	**0.005**	0.005
14	Bacillolysin	EC	BC_5351	Q814S1	65	**0.034**	0.013	**0.005**	0.006
15	Non-hemolytic enterotoxin NheA	EC [28]	BC_1809	Q81EZ8	44	**0.12**	0.05	**0.01**	0.01
16	Flagellin*	EC [101]	BC_1657-9	Q81FD3-5	29	**0.51**	0.08	**0.31**	0.10
	**more abundant proteins in the mutant**								
1	DNA-binding protein HU	C	BC_3728	Q81A62	10	**0.15**	0.02	**0.30**	0.02
2	Foldase protein PrsA 1	M	BC_1043	PRSA1_BACCR	32	**ND**		**0.010**	0.002
3	3-oxoacyl-[acyl-carrier-protein] synthase 2	M	BC_1174	Q81GL9	44	**ND**		**0.007**	0.001
4	50S ribosomal protein L10	C	BC_0119	RL10_BACCR	18	**0.009**	0.006	**0.065**	0.011
5	30S ribosomal protein S10	C	BC_0130	RS10_BACCR	12	**0.066**	0.016	**0.183**	0.029
6	DNA-binding protein HU	C	BC_1510	Q81FQ9	12	**0.44**	0.09	**0.75**	0.05
7	Elongation factor G	C	BC_0128	EFG_BACCR	76	**0.041**	0.010	**0.074**	0.005
8	30S ribosomal protein S11	C	BC_0157	RS11_BACCR	14	**0.005**	0.009	**0.077**	0.023
9	Putative triosephosphate isomerase	C	BC_5137	TPIS_BACCR	26	**0.005**	0.009	**0.063**	0.019
10	50S ribosomal protein L6	C	BC_0146	RL6_BACCR	20	**0.002**	0.004	**0.081**	0.028
11	Putative uncharacterized protein	U	BC_p0002	Q814F0	18	**0.10**	0.02	**0.43**	0.12
12	50S ribosomal protein L1	C	BC_0118	RL1_BACCR	25	**0.014**	0.009	**0.065**	0.019
13	50S ribosomal protein L3	C	BC_0131	RL3_BACCR	23	**0.001**	0.002	**0.021**	0.009
14	50S ribosomal protein L15	C	BC_0150	RL15_BACCR	15	**0.003**	0.005	**0.030**	0.011
15	Fructose-bisphosphate aldolase	C	BC_5335	Q814T5	31	**0.027**	0.008	**0.054**	0.010
16	50S ribosomal protein L21	C	BC_4438	RL21_BACCR	11	**0.085**	0.070	**0.225**	0.032
17	50S ribosomal protein L4	C	BC_0132	RL4_BACCR	23	**0.018**	0.007	**0.035**	0.006
18	30S ribosomal protein S15	C	BC_3806	RS15_BACCR	11	**0.022**	0.014	**0.067**	0.022

1 according to prediction of PSORTb algorithm (version 3.0.2; [26]): EC extracellular, C cytoplasmic, U unknown, M membrane; references for experimentally defined locations are given for proteins with predicted unknown localization.

2 **N**ormalized **S**pectral **A**bundance **F**actor, mean average of three biological replicates; the NSAF normalizes across samples and takes protein sizes into account; values range between 0 and 1, increasing values indicate higher abundance [27], stdev standard deviation of the means of three biological replicates; probability ranges associated with Students t-test (Scaffold 4.0.5).

3 ND not detected (NSAF 0 in at least two biological replicates and <0.005).

*due to high sequence similarity all peptide hits for “flagellin” (Q81FD3, Q81FD4, Q81FD5) were combined.

In Table 3 there is an error in the third footnote of the table caption. Please see the corrected Table 3 here.

**Table 3:** Genes with at least a five-fold differential transcription level in the ΔsecDF mutant compared to the isogenic wild type strain B. cereus ATCC 14579.

	Locus_tag^1^	Genbank_annotation	FC^2^	P – value^3^
***Resistance / Detoxification***	BC2984	Immune inhibitor A precursor	9.79	2.9E-07
	BC2985	Vancomycin B-type resistance protein vanW	8.63	1.6E-06
***Transport***	BC0816	periplasmic component of efflux system	5.01	2.9E-07
	BC3586	Oligopeptide-binding protein oppA	0.18	2.7E-03
	BC3788	Nucleoside transport system permease protein	0.06	2.9E-07
	BC3790	Nucleoside transport ATP-binding protein	0.11	3.5E-05
	BC3791	Nucleoside-binding protein	0.06	1.7E-06
	BC3792	Transcriptional regulator, GntR family	0.09	1.4E-05
	*BC4405*	*Protein translocase subunit SecDF*	0.13	1.0E-05
	BC4831	ABC transporter ATP-binding protein	6.68	3.9E-08
	BC5117	ABC transporter permease protein	0.11	1.6E-06
	BC5118	ABC transporter ATP-binding protein	0.12	3.1E-05
	BC5253	ABC transporter permease protein	0.08	9.1E-06
	BC5254	ABC transporter ATP-binding protein	0.11	5.0E-06
	BC5255	periplasmic component of efflux system	0.08	8.2E-07
***Metabolism***	BC0297	Guanine-hypoxanthine permease	0.08	9.2E-08
	BC0323§	PRAI carboxylase catalytic subunit	0.04	2.4E-08
	BC0324§	PRAI carboxylase ATPase subunit	0.07	2.1E-08
	BC0325§	Adenylosuccinate lyase	0.07	3.7E-07
	BC0326§	PRAI-succinocarboxamide synthase	0.04	2.4E-05
	BC0327§	PRFGA synthetase, PurS component	0.04	4.6E-06
	BC0328§	PRFGA synthase	0.04	1.9E-06
	BC0329§	PRFGA synthase	0.04	1.6E-06
	BC0330§	Amidophosphoribosyltransferase	0.04	4.8E-06
	BC0331§	PRFGA cyclo-ligase	0.04	6.2E-07
	BC0332§	Phosphoribosylglycinamide formyltransferase	0.05	1.6E-06
	BC0333§	IMP cyclohydrolase	0.06	6.1E-06
	BC0491	Formate acetyltransferase	0.18	2.1E-04
	BC0492	Pyruvate formate-lyase activating enzyme	0.15	7.7E-04
***Respiration***	BC1939	Cytochrome d ubiquinol oxidase subunit II	6.31	2.3E-05
	BC2119	Respiratory nitrate reductase beta chain	0.07	2.2E-04
	BC2120	Respiratory nitrate reductase delta chain	0.20	3.2E-02
	BC4792	Cytochrome d ubiquinol oxidase subunit I	0.14	8.6E-05
	BC4793	Cytochrome d ubiquinol oxidase subunit II	0.11	8.1E-04
***Putative Cell Wall Stress Response***	BC0813	enterotoxin / cell-wall binding protein entC	6.35	6.3E-07
	BC1435	hypothetical protein	33.96	2.1E-08
	BC1436	Phage shock protein A	12.83	7.9E-07
	BC5239	enterotoxin / cell-wall binding protein entA	5.60	7.9E-07
	BC5361	ECF-type sigma factor negative effector	12.40	1.7E-06
	BC5362	ECF-type sigma factor negative effector	8.26	2.4E-08
	BC5363	RNA polymerase ECF-type sigma factor	16.82	4.8E-07
***Motility***	BC1657	Flagellin	0.18	1.8E-06
	BC1659	Flagellin	0.19	6.7E-05
***Sigma B operon***	BC0862	Protease I	15.77	1.3E-05
	BC0863	Catalase	13.31	4.2E-06
	BC0998	General stress protein 17M	11.41	2.1E-08
	BC0999	hypothetical protein	12.27	2.8E-07
	BC1000	hypothetical Membrane Spanning Protein	12.54	6.7E-06
	BC1002	Anti-sigma B factor antagonist	5.36	2.5E-06
	BC1003	Anti-sigma B factor	8.97	1.4E-06
	BC1004	RNA polymerase sigma-B factor	7.84	1.8E-06
	BC1010	hypothetical protein	10.61	4.5E-06
	BC3130	hypothetical protein	5.30	7.4E-05
***Others***	BC0494	hypothetical Cytosolic Protein	0.19	6.7E-06
	BC1760	3-oxoacyl-[acyl-carrier-protein] synthase III	5.06	2.6E-06
	BC1852	Exonuclease SbcC	0.20	3.4E-04
	BC1854	hypothetical Cytosolic Protein	0.20	1.4E-04
	BC1861	DNA/RNA helicase (DEAD/DEAH box family)	0.20	3.2E-05
	BC2056	hypothetical protein	0.16	3.4E-07
	BC4482	hypothetical protein	5.32	6.5E-05
	BC4813	hypothetical protein	14.25	1.8E-07
	BC5116	hypothetical protein	0.16	1.3E-05
	BC5119	hypothetical protein	0.12	2.8E-05
	BC5120	hypothetical Cytosolic Protein	0.12	6.7E-06
	BC5121	hypothetical protein	0.12	1.7E-05
	BC5122	hypothetical Cytosolic Protein	0.18	2.4E-05
	BC5123	hypothetical protein	0.16	3.6E-05
	BC5124	hypothetical protein	0.19	2.7E-05
	BC5243	hypothetical protein	0.20	9.1E-05
	BC5252	hypothetical Membrane Spanning Protein	0.11	2.3E-06

1 data on the linear plasmid pBClin15 can be found in the supplementary file.

2 FC fold change of transcriptional expression in *B. cereus* Δ*secDF* compared to wild type.

3 P-values were computed using false discovery rate correction of 0.05 by an Bayesian linear model as integrated in the Limma-package [90]; data represent six independent cultures.

§ purine operon under the control of PurA.
