# Supplementary material for: SecDF as Part of the Sec-Translocase Facilitates Efficient Secretion of Bacillus cereus Toxins and Cell Wall-Associated Proteins
Source: PLoS One. 2014 Aug 1;9(8):e103326. doi: 10.1371/journal.pone.0103326 (PMC4118872; doi:10.1371/journal.pone.0103326)
Supplement: Figure S1 — Susceptibility of the ΔsecDF mutant towards selected compounds. (PDF) [file pone.0103326.s001.pdf]

Supplementary figure S1: Susceptibility of the Δ*secDF* mutant towards selected compounds.

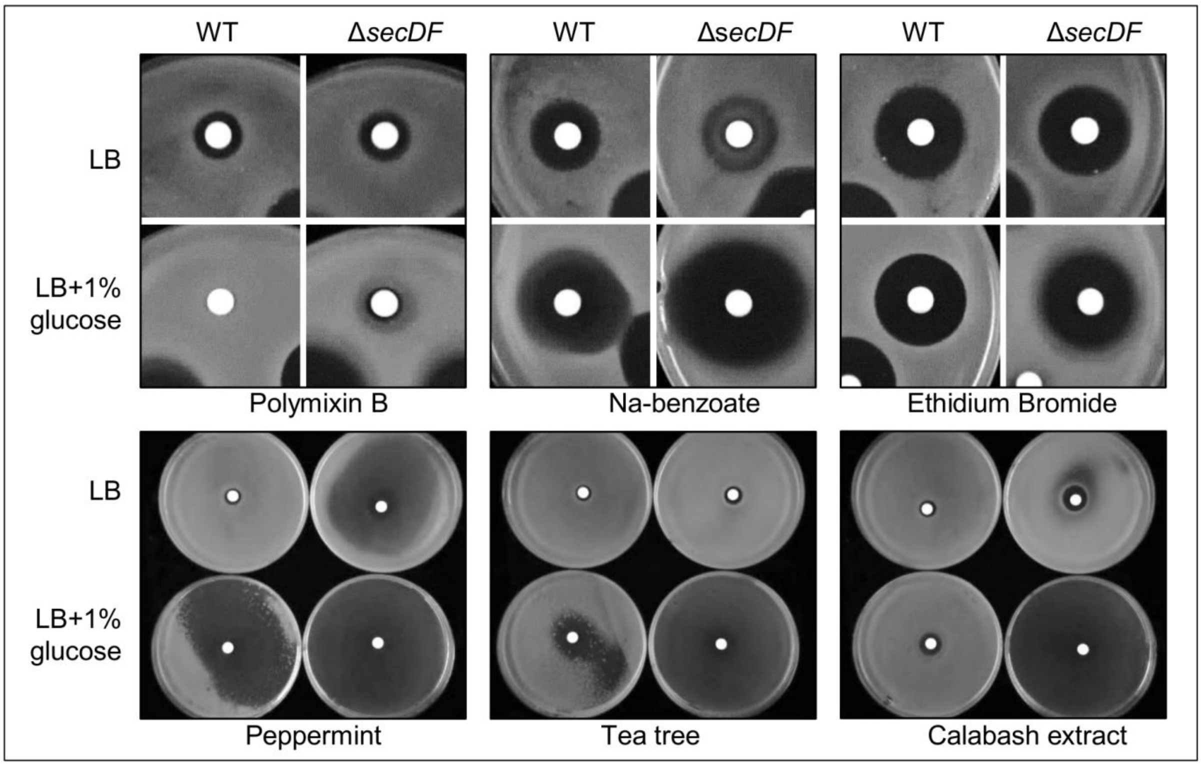

Suppl. figure S1: Susceptibility of *B. cereus* ATCC 14579 Δ*secDF* compared to the wild type to the indicated compounds measured by disk diffusion assays on LB +/- 1 % glucose. Bacteria were grown at 30 °C and all assays were performed 2-5 times independently.
