# Supplementary material for: SecDF as Part of the Sec-Translocase Facilitates Efficient Secretion of Bacillus cereus Toxins and Cell Wall-Associated Proteins
Source: PLoS One. 2014 Aug 1;9(8):e103326. doi: 10.1371/journal.pone.0103326 (PMC4118872; doi:10.1371/journal.pone.0103326)
Supplement: Figure S2 — Determination of lecithinase activity. (PDF) [file pone.0103326.s002.pdf]

Supplementary figure S2: Determination of lecithinase activity.

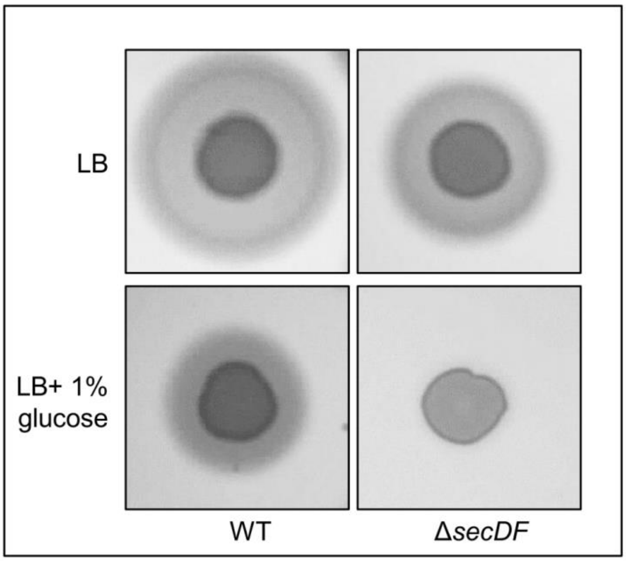

Suppl. figure S2: Determination of PC-PLC activity of actively growing cells. Bacteria were grown in LB over night at 30 °C, washed and incubated on LB supplemented with 5 % egg yolk +/- 1 % glucose for 7h at 30 °C. Similar results have been obtained by incubating the cells at 15 °C for 24h. The pictures are representative for three independent experiments.
