# Supplementary material for: SecDF as Part of the Sec-Translocase Facilitates Efficient Secretion of Bacillus cereus Toxins and Cell Wall-Associated Proteins
Source: PLoS One. 2014 Aug 1;9(8):e103326. doi: 10.1371/journal.pone.0103326 (PMC4118872; doi:10.1371/journal.pone.0103326)
Supplement: Figure S3 — Complementation of the ΔsecDF mutant. (PDF) [file pone.0103326.s003.pdf]

Supplementary figure S3: Complementation of the Δ*secDF* mutant.

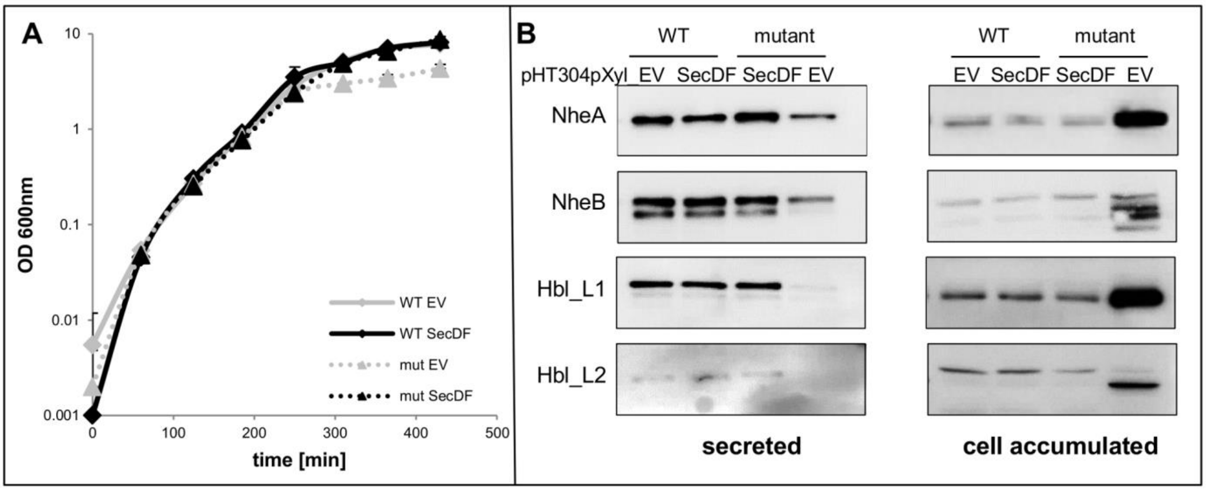

Suppl. figure S3: Complementation of the Δ*secDF* mutant restores both growth and toxin secretion.

A) Cells were grown in LBG supplemented with erythromycin 5µg/ml and 20mM xylose. Each point is the mean of two biological replicates and error bars indicate the standard deviation.

B) Western blot analyses of toxin components in the growth medium (left) and cell-associated (right). Expression of SecDF encoded on pHT304pXyl was induced by 20mM xylose. The growth medium and the cells were collected after 4h of culture. Strains: WT EV: wild type strain with pHT304*Pxyl*; WT SecDF: wild type strain with pHT304*Pxyl_secDF*; mutant EV: Δ*secDF* strain with pHT304*Pxyl*; mutant SecDF: Δ*secDF* strain with pHT304*Pxyl_secDF*

The blots are representative for the results obtained for two independent experiments.
