# Supplementary material for: SecDF as Part of the Sec-Translocase Facilitates Efficient Secretion of Bacillus cereus Toxins and Cell Wall-Associated Proteins
Source: PLoS One. 2014 Aug 1;9(8):e103326. doi: 10.1371/journal.pone.0103326 (PMC4118872; doi:10.1371/journal.pone.0103326)
Supplement: Figure S4 — Validation of microarray results by qRT-PCR. (PDF) [file pone.0103326.s004.pdf]

Supplementary figure S4: Validation of microarray results by qRT-PCR.

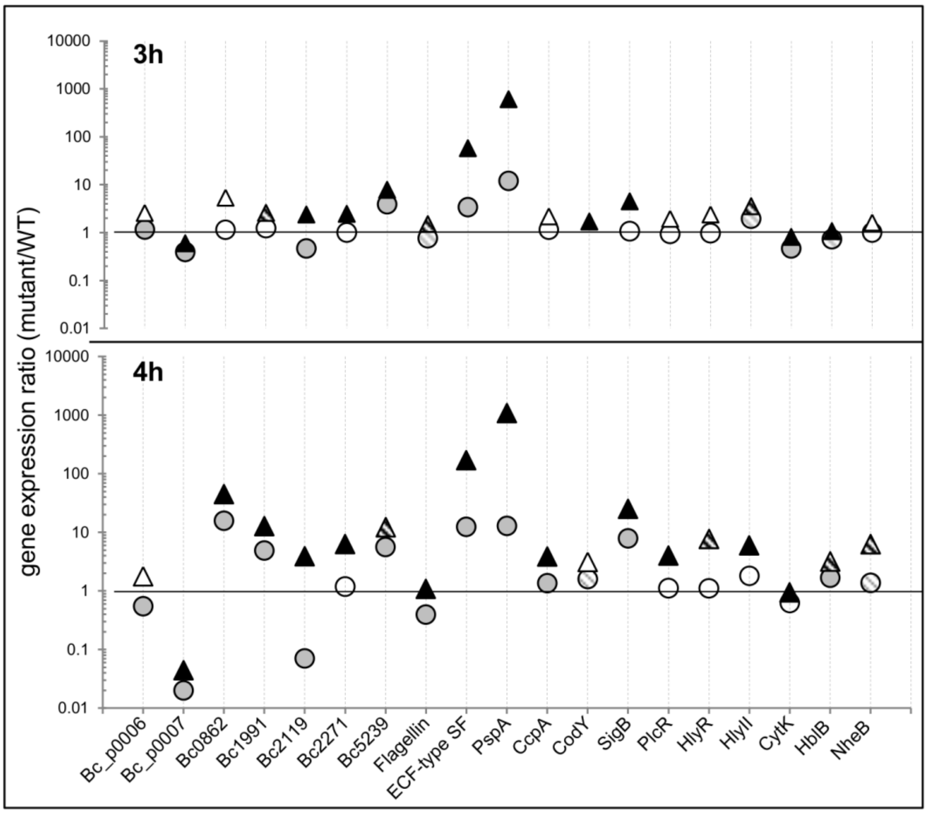

Suppl. Figure S4: Validation of microarray results by quantitative RT-PCR. Shown are the gene expression ratios (WT/Δ*secDF*) for selected genes of the microarray results (circles) and the qRT-PCR (triangles) at 3h and 4h growth in LBG at 30 °C. The 3h and 4h microarrays represent two and six biological replicates, respectively. The qRT-PCR data derive from two biological replicates, independent from the ones used for the microarray trials. Filled markers ($p<0.05$), patterned markers ($p<0.15$), non-filled markers ($p>0.15$) represent probability ranges derived from Bayesian linear modelling using the limma-package (microarray) or which associated with a two-tailed, paired Students t-test (q-RT-PCR).
