# Supplementary material for: SecDF as Part of the Sec-Translocase Facilitates Efficient Secretion of Bacillus cereus Toxins and Cell Wall-Associated Proteins
Source: PLoS One. 2014 Aug 1;9(8):e103326. doi: 10.1371/journal.pone.0103326 (PMC4118872; doi:10.1371/journal.pone.0103326)
Supplement: Figure S5 — Regulation of pBClin15 ORFs. (PDF) [file pone.0103326.s005.pdf]

Supplementary figure S5: Regulation of pBClin15 ORFs.

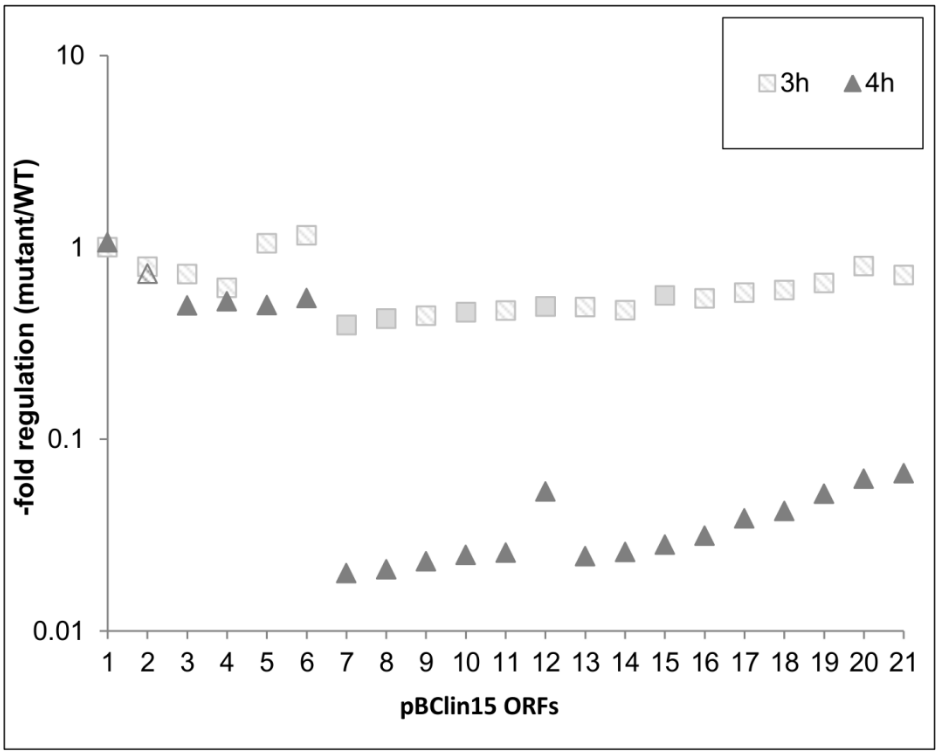

Suppl. figure S5: Representation of the bipartite gene regulation of ORF 1-6 and ORF 7-21 of the linear plasmid pBClin15 in *B. cereus* ATCC 14579. Microarray results are derived from two (3h, squares) and six (4h, triangles) biological replicates, respectively. 1–fold regulation means no differential gene expression, while 0.1-fold describes 10-times downregulation in the Δ*secDF* mutant. Filled markers (*P-values* <0.05), patterned markers (*P*-values >0.05) represent probability ranges derived from Bayesian linear modelling using the limma-package.
