# Supplementary material for: SecDF as Part of the Sec-Translocase Facilitates Efficient Secretion of Bacillus cereus Toxins and Cell Wall-Associated Proteins
Source: PLoS One. 2014 Aug 1;9(8):e103326. doi: 10.1371/journal.pone.0103326 (PMC4118872; doi:10.1371/journal.pone.0103326)
Supplement: Table S1 — Susceptibility to toxic compounds of B. cereus ATCC 14579 wild type strain and its isogenic ΔsecDF variant. (PDF) [file pone.0103326.s006.pdf]

Supplemental tables

Table S1: Susceptibility to toxic compounds of *B. cereus* ATCC 14579 wild type strain and its isogenic Δ*secDF* variant.

	Microdilution broth assay						Disk diffusion assay	
**Compound**	**LB**			**LB + 1% glucose**			**LB**	**LB + 1% glucose**
[µg/ml]	WT	Δ*secDF*	FD[1]	WT	Δ*secDF*	FD		
Oxytetracycline	4	4*	1*			ND	-	(+)
Chlorhexidine	4	2	2	4	2	2	-	-
Polymyxin B	800	400-800	1-2	800	400	2	-	+
Gentamicin	12.5	6.25	2	12.5	3.2	4	§	§
Ampicillin	1000	500	2	1000	500-1000	1-2	-	-
SDS	0.01	0.005	2	0.01	0.0025	4	-	-
DOC	0.4	0.2	2	0.2	0.2*	1*	-	-
Na-Benzoate[2]	6.25-12.5	6.25*	1-2*	6.25	1.6	4	-	+

* means the mutant strain grew to the same MIC values as the WT strain, but growth density was visibly reduced

[1] FD -fold difference in susceptibility

[2] in mg/ml

Microdilution broth assays revealing no differences in compound susceptibility when grown in LB: erythromycin, tetracycline, alexidine, cetyltrimethylammonium bromide, cetylpyridinium chloride, domiphen bromide, chlorpromazine, dichlofluanide, K-lactate, Na-lactate, CCCP

All conditions were tested with at least two biological replicates and with two technical replicates each.

Disk diffusion tests revealed, in at least two independent experiments: - no differences of growth inhibition zone diameter between mutant and WT strain; (+) diffused inhibition zone of the mutant strain compared to a clear inhibition zone of the WT strain; + bigger inhibition zone of the mutant strain due to decreased resistance,

Disk diffusion tests revealing no differences in compound susceptibility: oxacillin, chloramphenicol, phosphomycin, ciprofloxacin, desoxycholate, vancomycin; § no stable results were obtained using gentamicin impregnated disks
