# Supplementary material for: SecDF as Part of the Sec-Translocase Facilitates Efficient Secretion of Bacillus cereus Toxins and Cell Wall-Associated Proteins
Source: PLoS One. 2014 Aug 1;9(8):e103326. doi: 10.1371/journal.pone.0103326 (PMC4118872; doi:10.1371/journal.pone.0103326)
Supplement: Table S2 — Susceptibility to toxic compounds of E. coli BW25113_ΔacrB expressing SecDF. (PDF) [file pone.0103326.s007.pdf]

Table S2:
Susceptibility to toxic compounds of *E. coli* BW25113_Δ*acrB* expressing SecDF.

		MIC (µg/ml)					
		uninduced			0.05mM IPTG		
Compound class	compound	EV	SecDF	FD	EV	SecDF	FD
Aminocoumarin	Novobicin	2-4	2-4	1			ND
Aminoglycoside	Gentamycin	9	9	1	9	9	1
Fusidane	Fusidic Acid	24	3-6	0.25			ND
Macrolide	Erythromycin	8	8	1	8	8	1
Other antibiotics	Chloramphenicol	1	1	1	1	1	1
	Tetracycline	1	1	1	1	1	1
	Oxytetracycline	2	2	1	2	2	1
Detergents	Bile Salt	5000-10000	5000-10000	1	>10000	>10000	
	DOC	2500-5000	5000	1			
	SDS	100	100	1	100	100	1
Stains	Acriflavine	25	12.5	0.5			ND
	Crystal Violet	0.5	0.5	1	0.25	0.25	1
	Ethidium Bromide	10	10	1	10	10	1
	Rhodamine 6G	4.7-9.4	4.7-9.4	1			ND
Quaternary ammonium salt	Berberine	128	128	1			ND
Other	TPP+	12.5	12.5	1			
	**Sodium benzoate**	**6250**	**12500**	**2**	**6250**	**12500**	**2**
	Sodium lactate	5	5	1	5	5	1
	Chlorhexidin	0.2	0.2	1	0.2	0.2	1
Plant extracts (%)	Peppermint	0.2	0.2	1	0.2	0.2	1
	Cajute	0.45	0.45	1	0.45	0.45	1
	Tea tree	0.63	0.63-1.25	1	0.63-1.25	0.63	1

Minimum inhibition concentrations were determined using the microdilution method with 2-fold dilutions. *E. coli* strains carrying pTTQ18 with (“SecDF”) or without (“EV” empty vector) *secDF* were grown in LB supplemented with 50µg/ml ampicillin until mid-exponential phase. Using these pre-cultures 96-well plates containing LB with 50µg/ml ampicillin were inoculated to a final density of OD600nm 0.02 in a volume of 100µl. Protein expression was induced with 0.05mM IPTG. After 22h incubation at 30 °C, 220rpm the MIC value was set to the lowest concentration of no visible bacterial growth. All conditions were tested with at least two biological replicates and with two technical replicates each.
FD, fold difference; ND, not determined; DOC, Deoxycholate; SDS, Sodium Dodecylsulfate; TPP+, tetraphenylphosphonium ion
