# Supplementary material for: SecDF as Part of the Sec-Translocase Facilitates Efficient Secretion of Bacillus cereus Toxins and Cell Wall-Associated Proteins
Source: PLoS One. 2014 Aug 1;9(8):e103326. doi: 10.1371/journal.pone.0103326 (PMC4118872; doi:10.1371/journal.pone.0103326)
Supplement: Table S3 — Secretome of B. cereus ATCC 14579 wild type and ΔsecDF mutant. (PDF) [file pone.0103326.s008.pdf]

Table S3: Secretome of *B. cereus* ATCC 14579 wild type and Δ*secDF* mutant.

#	Identified Proteins	Locali-zation[1]	locus tag	Uniprot Acc. Nr.	MW (kDa)	P-value[2]	WT NSAF avg[3]	WT stdev	Δ*secDF* NSAF avg	Δ*secDF* stdev
1	Cold shock protein	C	BC_3539	Q81AM3	7	0.11	**0.58**	0.07	**0.25**	0.26
2	Phospholipase C	EC	BC_0670	Q81HW1	32	0.0015	**0.55**	0.12	**0.00**	0.00
3	Flagellin*	EC [1]	BC_1657-59	Q81FD3-5	29	0.049	**0.51**	0.08	**0.31**	0.10
4	DNA-binding protein HU	C	BC_1510	Q81FQ9	12	0.0064	**0.44**	0.09	**0.75**	0.05
5	Enterotoxin	EC [2]	BC_1953	Q81EL6	45	0.1	**0.38**	0.07	**0.20**	0.12
6	Sphingomyelin phosphodiesterase	EC	BC_0671	Q81HW0	37	0.012	**0.28**	0.11	**0.00**	0.00
7	50S ribosomal protein L7/L12	C	BC_0120	RL7_BACCR	13	0.46	**0.28**	0.09	**0.33**	0.08
8	Cold shock-like protein CspD	C	BC_4859	CSPD_BACCR	7	0.8	**0.26**	0.06	**0.23**	0.20
9	Hemolysin BL lytic component L2	EC [3]	BC_3104	Q81BP7	49	0.0066	**0.25**	0.08	**0.00**	0.00
10	Non-hemolytic enterotoxin NheB	EC	BC_1810	Q81EZ7	43	0.0026	**0.21**	0.05	**0.01**	0.01
11	Enterotoxin / cell-wall binding pr. EntC	EC [2]	BC_0813	Q81HJ4	46	0.14	**0.19**	0.08	**0.47**	0.25
12	Hemolysin BL lytic component L1	EC	BC_3103	Q7BYC6	44	0.0055	**0.18**	0.05	**0.01**	0.01
13	DNA-binding protein HU	C	BC_3728	Q81A62	10	0.001	**0.15**	0.02	**0.30**	0.02
14	Cytotoxin K	EC	BC_1110	Q81GS6	37	< 0.0001	**0.14**	0.01	**0.00**	0.00
15	Major cold shock protein CspA	C	BC_1131	CSPA_BACCR	7	0.91	**0.14**	0.05	**0.13**	0.15
16	Non-hemolytic enterotoxin NheA	EC [2]	BC_1809	Q81EZ8	44	0.033	**0.12**	0.05	**0.01**	0.01
17	Flagellar hook-associated protein 3	EC [2]	BC_1637	Q81FF3	32	0.078	**0.10**	0.01	**0.05**	0.04
18	30S ribosomal protein S7	C	BC_0126	RS7_BACCR	18	0.3	**0.10**	0.04	**0.14**	0.03
19	Hemolysin BL binding component	EC	BC_3102	Q81BP9	42	0.022	**0.10**	0.05	**0.00**	0.00
20	Putative uncharacterized protein	U	BC_p0002	Q814F0	18	0.0099	**0.10**	0.02	**0.43**	0.12
21	50S ribosomal protein L11-1	C	BC_0117	RL11A_BACCR	15	0.31	**0.10**	0.05	**0.06**	0.04
22	50S ribosomal protein L2	C	BC_0134	RL2_BACCR	30	0.59	**0.095**	0.032	**0.117**	0.059
23	Microbial collagenase	EC	BC_0556	Q81I63	109	0.022	**0.088**	0.039	**0.005**	0.005
24	50S ribosomal protein L21	C	BC_4438	RL21_BACCR	11	0.035	**0.085**	0.070	**0.225**	0.032

25	N-acetylmuramoyl-L-alanine amidase	EC [2]	BC_5234	Q815D0	61	0.56	**0.085**	0.021	**0.065**	0.050
26	Pyruvate dehydrogenase E1 c. β-SU	C	BC_3972	Q819K4	35	0.58	**0.082**	0.026	**0.073**	0.003
27	50S ribosomal protein L27	C	BC_4436	RL27_BACCR	11	0.51	**0.078**	0.058	**0.044**	0.057
28	N-acetylmuramoyl-L-alanine amidase	CW	BC_5196	Q815G3	48	0.86	**0.078**	0.012	**0.072**	0.052
29	Elongation factor Ts	C	BC_3824	EFTS_BACCR	32	0.25	**0.072**	0.015	**0.086**	0.011
30	50S ribosomal protein L24	C	BC_0142	RL24_BACCR	11	0.78	**0.071**	0.016	**0.061**	0.055
31	Enterotoxin / cell-wall binding pr. EntA	EC	BC_5239	Q815C5	31	0.088	**0.070**	0.037	**0.348**	0.212
32	Elongation factor Tu	C	BC_0129	EFTU_BACCR	43	0.42	**0.066**	0.012	**0.091**	0.047
33	30S ribosomal protein S10	C	BC_0130	RS10_BACCR	12	0.0037	**0.066**	0.016	**0.183**	0.029
34	Dihydrolipoyl dehydrogenase	C	BC_3970	Q819K6	49	0.52	**0.063**	0.015	**0.070**	0.009
35	Flagellar hook-associated protein 1	EC [2]	BC_1636	Q81FF4	48	0.06	**0.061**	0.032	**0.011**	0.008
36	50S ribosomal protein L30	C	BC_0149	RL30_BACCR	7	0.054	**0.050**	0.025	**0.113**	0.031
37	10 kDa chaperonin	C	BC_0294	CH10_BACCR	10	0.51	**0.043**	0.038	**0.082**	0.087
38	Elongation factor G	C	BC_0128	EFG_BACCR	76	0.0064	**0.041**	0.010	**0.074**	0.005
39	Antigen	U	BC_3699	Q81A83	36	0.061	**0.041**	0.007	**0.142**	0.067
40	50S ribosomal protein L5	C	BC_0143	RL5_BACCR	20	0.094	**0.040**	0.015	**0.105**	0.049
41	Ribosome-recycling factor	C	BC_3822	RRF_BACCR	21	0.12	**0.038**	0.001	**0.050**	0.010
42	30S ribosomal protein S9	C	BC_0165	RS9_BACCR	14	0.26	**0.037**	0.031	**0.081**	0.048
43	Trigger factor	C	BC_4480	TIG_BACCR	47	0.16	**0.037**	0.011	**0.078**	0.041
44	Cell wall-binding protein	EC	BC_0679	Q81HV2	47	0.27	**0.037**	0.011	**0.019**	0.020
45	Bacillolysin	EC	BC_5351	Q814S1	65	0.027	**0.034**	0.013	**0.005**	0.006
46	Microbial collagenase Sfp	C	BC_3762	Q81A31	67	0.054	**0.029**	0.019	**0.000**	0.000
47	Enterotoxin / cell-wall binding pr. EntB	EC [2]	BC_2952	Q81C32	55	0.00023	**0.028**	0.004	**0.001**	0.001
48	Putative murein endopeptidase	U	BC_1991	Q81EI5	44	0.003	**0.028**	0.007	**0.000**	0.000
49	Fructose-bisphosphate aldolase	C	BC_5335	Q814T5	31	0.023	**0.027**	0.008	**0.054**	0.010
50	Enolase	C	BC_5135	ENO_BACCR	46	0.15	**0.026**	0.009	**0.041**	0.011
51	Pyruvate dehydrogenase E1 c. α-SU	C	BC_3973	Q819K3	41	0.076	**0.026**	0.015	**0.077**	0.034
52	Glyceraldehyde 3phosphate dehydrogenase	C	BC_5140	Q815K6	35	0.23	**0.026**	0.004	**0.054**	0.035
53	Flagellar hook protein flgE	EC	BC_1651	Q81FE1	46	0.12	**0.025**	0.021	**0.001**	0.002

54	Dihydrolipoamide acetyltransferase	C	BC_3971	Q819K5	46	0.2	**0.023**	0.024	**0.048**	0.015
55	Superoxide dismutase [Mn] 1	EC	BC_4272	SODM1_BACCR	25	0.64	**0.023**	0.008	**0.028**	0.016
56	30S ribosomal protein S15	C	BC_3806	RS15_BACCR	11	0.04	**0.022**	0.014	**0.067**	0.022
57	Cell wall endopeptidase, family M23/M37	U	BC_3698	Q81A84	30	0.33	**0.022**	0.016	**0.036**	0.015
58	Flagellar hook-associated protein 2	EC	BC_1638	Q81FF2	51	0.35	**0.019**	0.013	**0.008**	0.013
59	50S ribosomal protein L4	C	BC_0132	RL4_BACCR	23	0.037	**0.018**	0.007	**0.035**	0.006
60	30S ribosomal protein S13	C	BC_0156	RS13_BACCR	14	0.13	**0.018**	0.013	**0.045**	0.021
61	Cell wall endopeptidase, family M23/M37	EC	BC_0740	Q81HR4	42	0.017	**0.016**	0.006	**0.001**	0.002
62	Translation initiation factor IF-1	C	BC_0154	IF1_BACCR	8	0.12	**0.016**	0.014	**0.000**	0.000
63	50S ribosomal protein L17	C	BC_0159	RL17_BACCR	13	0.12	**0.015**	0.020	**0.062**	0.036
64	50S ribosomal protein L1	C	BC_0118	RL1_BACCR	25	0.014	**0.014**	0.009	**0.065**	0.019
65	Hypothetical cytosolic protein	U	BC_p0018	Q814D4	29	0.38	**0.014**	0.009	**0.007**	0.007
66	30S ribosomal protein S5	C	BC_0148	RS5_BACCR	18	0.084	**0.011**	0.006	**0.073**	0.047
67	50S ribosomal protein L14	C	BC_0141	RL14_BACCR	13	0.38	**0.009**	0.011	**0.022**	0.020
68	Chitin binding protein	CW	BC_2798	Q81CG6	50	0.28	**0.009**	0.012	**0.000**	0.000
69	50S ribosomal protein L10	C	BC_0119	RL10_BACCR	18	0.0016	**0.009**	0.006	**0.065**	0.011
70	Enoyl-[acyl-carrier-protein] reductase [NADH] FabI	M	BC_1216	FABI_BACCR	28	0.2	**0.008**	0.007	**0.002**	0.003
71	Hypoxanthine-guanine phosphoribosyltransferase	C	BC_0071	Q81J83	20	0.87	**0.005**	0.005	**0.004**	0.007
72	Putative triosephosphate isomerase	C	BC_5137	TPIS_BACCR	26	0.0081	**0.005**	0.009	**0.063**	0.019
73	30S ribosomal protein S11	C	BC_0157	RS11_BACCR	14	0.0068	**0.005**	0.009	**0.077**	0.023
74	Alkyl hydroperoxide reductase C22	C	BC_0377	Q81IK9	21	0.87	**0.005**	0.004	**0.006**	0.011
75	Hypothetical cytosolic protein	U	BC_p0010	Q814E2	40	0.9	**0.005**	0.002	**0.004**	0.005
76	Ferrichrome-binding protein	M	BC_5380	Q814P4	31	0.12	**0.005**	0.004	**0.000**	0.000
77	60 kDa chaperonin	C	BC_0295	CH60_BACCR	57	0.36	**0.004**	0.004	**0.009**	0.008
78	50S ribosomal protein L19	C	BC_3838	RL19_BACCR	13	0.59	**0.004**	0.007	**0.010**	0.018
79	putative murein endopeptidase	CW	BC_0991	Q81H34	65	0.014	**0.004**	0.001	**0.000**	0.000
80	Chaperone protein DnaK	C	BC_4312	DNAK_BACCR	66	0.17	**0.004**	0.002	**0.013**	0.009
81	Perfringolysin O	EC	BC_5101	Q815P0	57	0.00027	**0.004**	0.001	**0.000**	0.000

82	50S ribosomal protein L13	C	BC_0164	RL13_BACCR	16	0.2	**0.003**	0.005	**0.021**	0.020
83	50S ribosomal protein L15	C	BC_0150	RL15_BACCR	15	0.021	**0.003**	0.005	**0.030**	0.011
84	Glucose-6-phosphate isomerase	C	BC_4898	G6PI_BACCR	50	0.69	**0.003**	0.001	**0.004**	0.003
85	50S ribosomal protein L6	C	BC_0146	RL6_BACCR	20	0.0092	**0.002**	0.004	**0.081**	0.028
86	NADP-dependent glyceraldehyde-3-phosphate dehydrogenase	C	BC_0868	Q81HE6	52	0.059	**0.002**	0.002	**0.012**	0.006
87	30S ribosomal protein S6	C	BC_5476	RS6_BACCR	11	0.062	**0.002**	0.004	**0.076**	0.050
88	30S ribosomal protein S4	C	BC_4655	RS4_BACCR	23	0.2	**0.002**	0.003	**0.018**	0.019
89	50S ribosomal protein L3	C	BC_0131	RL3_BACCR	23	0.018	**0.001**	0.002	**0.021**	0.009
90	N-acetylmuramoyl-L-alanine amidase	CW	BC_0902	Q81HB4	59	0.37	**0.001**	0.002	**0.000**	0.000
91	30S ribosomal protein S3	C	BC_0137	RS3_BACCR	24	0.13	**0.000**	0.000	**0.013**	0.012
92	Foldase protein PrsA 1	M	BC_1043	PRSA1_BACCR	32	0.0014	**0.000**	0.000	**0.010**	0.002
93	3-oxoacyl-[acyl-carrier-protein] synthase 2	M	BC_1174	Q81GL9	44	0.0014	**0.000**	0.000	**0.007**	0.001
94	Phosphoglycerol transferase	M	BC_5232	Q815D2	72	0.19	**0.000**	0.000	**0.005**	0.006
95	Iron(III) dicitrate-binding protein	M	BC_3738	Q81A53	36	0.37	**0.000**	0.000	**0.004**	0.006
96	Internalin	CW	BC_1331	Q81G77	86	0.37	**0.000**	0.000	**0.001**	0.002

[1] according to prediction of PSORTb algorithm (version 3.0.2; [4]): EC extracellular, C cytoplasmic, U unknown, M membrane; references for experimentally defined locations are given for proteins with predicted unknown localization

[2] probability ranges associated with a Students t-test (Scaffold 4.0.5)

[3] **N**ormalized **S**pectral **A**bundance **F**actor, mean average of three biological replicates; the NSAF normalizes across samples and takes protein sizes into account; values range between 0 and 1, increasing values indicate higher abundance [5], stdev standard deviation of the means of three biological replicates

* due to high sequence similarity all peptide hits for “flagellin” (Q81FD3, Q81FD4, Q81FD5) were combined

References:

1. LaVallie ER, Stahl ML (1989) Cloning of the flagellin gene from Bacillus subtilis and complementation studies of an in vitro-derived deletion mutation. Journal of Bacteriology 171: 3085-3094.
2. Clair G, Roussi S, Armengaud J, Duport C (2010) Expanding the Known Repertoire of Virulence Factors Produced by Bacillus cereus through Early Secretome Profiling in Three Redox Conditions. Molecular & Cellular Proteomics 9: 1486-1498.
3. Beecher DJ, Schoeni JL, Wong AC (1995) Enterotoxic activity of hemolysin BL from Bacillus cereus. Infection and Immunity 63: 4423-4428.
4. Yu NY, Wagner JR, Laird MR, Melli G, Rey S, et al. (2010) PSORTb 3.0: improved protein subcellular localization prediction with refined localization subcategories and predictive capabilities for all prokaryotes. Bioinformatics 26: 1608-1615.
5. Zybailov B, Mosley AL, Sardiu ME, Coleman MK, Florens L, et al. (2006) Statistical Analysis of Membrane Proteome Expression Changes in Saccharomyces cerevisiae. Journal of Proteome Research 5: 2339-2347.
