# Supplementary material for: SecDF as Part of the Sec-Translocase Facilitates Efficient Secretion of Bacillus cereus Toxins and Cell Wall-Associated Proteins
Source: PLoS One. 2014 Aug 1;9(8):e103326. doi: 10.1371/journal.pone.0103326 (PMC4118872; doi:10.1371/journal.pone.0103326)
Supplement: Table S4 — Transcriptional activation of the SigB regulon in the B. cereus ATCC 14579 ΔsecDF mutant compared to its wild type strain. (PDF) [file pone.0103326.s009.pdf]

Table S4:
Transcriptional activation of the SigB regulon in the *B. cereus* ATCC 14579 Δ*secDF* mutant compared to its wild type strain.

		4h Δ*secDF* vs. WT	Heat shock WT 42C/30C 10min[1]
Bc0862	Protease I	**15.8**	72.9
katE	Catalase	**13.3**	34.0
BC0995	hypothetical protein	1.3	2.2
BC0996	hypothetical protein	1.2	2.0
BC0998	General stress protein 17M	**11.4**	70.9
BC0999	hypothetical protein	**12.3**	ND
BC1000	hypothetical membrane spanning protein	**12.5**	36.3
BC1001	hypothetical protein	**2.0**	35.8
rsbV	Anti-sigma B factor antagonist	**5.4**	38.3
rsbW	Anti-sigma B factor	**9.0**	52.4
SigB	RNA polymerase sigma-B factor	**7.8**	28.1
orf4	Bacterioferritin	ND	56.0
rsbY	Sigma factor sigB regulation protein rsbU	**1.8**	5.1
BC1007	Chemotaxis protein methyltransferase	1.3	3.6
BC1008	Two component system histidine kinase	1.4	4.4
BC1009	hypothetical protein	**1.8**	87.5
BC1010	hypothetical protein	**10.6**	5.5
BC1011	hypothetical protein	4.3	23.7
BC1012	hypothetical protein	2.5	ND
sigE	RNA polymerase ECF-type sigma factor	1.2	3.8
BC2638	Spore germination protein LC	0.9	4.3
BC3130	hypothetical protein	**5.3**	10.3
BC3131	hypothetical protein	**2.4**	42.6
BC3132	General stress protein 17M	**4.1**	2.0
BC4641	hypothetical protein	1.2	3.8
gerQ	hypothetical protein	0.7	6.1

[1] Comparison of SigB regulon stimulation by growth in LBG for 4h and by heat shock as shown by Van Schaik *et al.* [1]

Bold numbers represent transcriptional changes with FDR – corrected p-values < 0.02 as determined by Bayesian linear modelling using the Limma-package.

Reference:

1. van Schaik W, van der Voort M, Molenaar D, Moezelaar R, de Vos WM, et al. (2007) Identification of the σB Regulon of Bacillus cereus and Conservation of σB-Regulated Genes in Low-GC-Content Gram-Positive Bacteria. Journal of Bacteriology 189: 4384-4390.
