# Supplementary material for: SecDF as Part of the Sec-Translocase Facilitates Efficient Secretion of Bacillus cereus Toxins and Cell Wall-Associated Proteins
Source: PLoS One. 2014 Aug 1;9(8):e103326. doi: 10.1371/journal.pone.0103326 (PMC4118872; doi:10.1371/journal.pone.0103326)
Supplement: Table S5 — Complete list of microarray results. The list shows at least 2-fold differentially regulated genes in the B. cereus ATCC 1459 ΔsecDF mutant compared to wild type (P-value <0.05). (PDF) [file pone.0103326.s010.pdf]

Table S5: Complete list of microarray results.

		3h time point		4h time point	
**Locus_tag**	**Genbank_annotation**	**FC[1]**	**P-Value[2]**	**FC**	**adj.P.Val[3]**
BC_p0003	putative cytoplasmic protein	0.73	*1.4E-01*	0.50	2.1E-04
BC_p0007	hypothetical protein	0.39	3.2E-02	0.02	3.6E-09
BC_p0008	putative cytoplasmic protein	0.43	3.5E-02	0.02	5.6E-08
BC_p0009	putative cytoplasmic protein	0.44	*5.5E-02*	0.02	2.3E-07
BC_p0010	putative cytoplasmic protein	0.46	3.5E-02	0.02	2.1E-08
BC_p0011	hypothetical protein	0.47	*7.6E-02*	0.03	2.2E-07
BC_p0012	hypothetical protein	0.49	2.2E-02	0.05	1.5E-08
BC_p0013	putative cytoplasmic protein	0.49	*5.7E-02*	0.02	1.9E-09
BC_p0014	putative cytoplasmic protein	0.47	2.6E-02	0.03	6.7E-08
BC_p0015	hypothetical protein	0.56	*9.8E-02*	0.03	1.9E-09
BC_p0016	N-acetylmuramoyl-L-alanine amidase	0.54	*5.2E-02*	0.03	6.7E-09
BC_p0017	hypothetical protein	0.58	*6.9E-02*	0.04	1.9E-09
BC_p0018	putative cytoplasmic protein	0.60	*1.1E-01*	0.04	3.6E-09
BC_p0019	hypothetical protein	0.65	*1.1E-01*	0.05	1.9E-09
BC_p0020	hypothetical protein	0.80	*3.3E-01*	0.06	6.0E-09
BC_p0021	N-acetylmuramoyl-L-alanine amidase	0.72	*1.3E-01*	0.07	1.9E-08
BC0001	Chromosomal replication initiator protein dnaA	1.45	3.9E-02	0.48	1.5E-03
BC0013	Inosine-5'-monophosphate dehydrogenase	1.02	*9.1E-01*	3.36	1.8E-06
BC0016	pyridoxine biosynthesis amidotransferase	0.64	*8.8E-02*	0.49	4.5E-03
BC0101	Arginine kinase	2.42	*8.4E-02*	2.24	1.1E-04
BC0102	Neg. regulator of genetic competence clpC/mecB	1.98	3.3E-02	2.15	4.9E-04
BC0109	Serine acetyltransferase	0.86	*4.5E-01*	0.46	2.1E-04
BC0121	16S rRNA m(2)G 1207 methyltransferase	1.14	*5.8E-01*	0.41	2.8E-04
BC0159	LSU ribosomal protein L17P	0.96	*7.8E-01*	0.46	4.5E-04
BC0159	LSU ribosomal protein L17P	0.99	*9.6E-01*	0.48	3.4E-04
BC0160	Cobalt transport ATP-binding protein cbiO	0.90	*4.7E-01*	0.46	6.2E-04
BC0161	Cobalt transport ATP-binding protein cbiO	0.83	*3.9E-01*	0.44	6.3E-03
BC0162	Cobalt transport protein cbiQ	0.74	*5.5E-01*	0.37	2.7E-04
BC0163	tRNA pseudouridine synthase A	ND	ND	0.33	4.6E-05
BC0194	hypothetical Membrane Spanning Protein	0.15	6.6E-03	0.29	1.7E-04
BC0218	Pyrroline-5-carboxylate reductase	1.10	*5.7E-01*	2.28	1.2E-04
BC0223	hypothetical protein	1.53	3.4E-02	2.11	1.4E-04
BC0226	hypothetical protein	0.94	*7.8E-01*	0.44	4.5E-04
BC0227	hypothetical protein	1.89	*6.2E-02*	3.42	7.6E-06
BC0236	Transglycosylase	ND	ND	2.03	5.9E-04
BC0297	Guanine-hypoxanthine permease	1.58	*1.1E-01*	0.08	9.2E-08
BC0304	FrnE protein	1.09	*6.5E-01*	2.58	2.6E-05
BC0323	Phosphoribosylaminoimidazole carboxylase cat. SU	0.91	*5.9E-01*	0.04	2.4E-08
BC0324	Phosphoribosylaminoimidazole carboxylase ATPase SU	0.82	*4.2E-01*	0.07	2.1E-08
BC0325	Adenylosuccinate lyase	0.72	*1.8E-01*	0.07	3.7E-07
BC0326	Phosphoribosylamidoimidazole-succinocarboxamide S.	0.79	*1.6E-01*	0.04	2.4E-05

BC0327	Phosphorybosylformylglycinamidine synthetase	0.87	*6.4E-01*	0.04	4.6E-06
BC0328	Phosphoribosylformylglycinamidine synthase	0.77	*2.8E-01*	0.04	1.9E-06
BC0329	Phosphoribosylformylglycinamidine synthase	0.85	*4.9E-01*	0.04	1.6E-06
BC0330	Amidophosphoribosyltransferase	0.85	*3.7E-01*	0.04	4.8E-06
BC0331	Phosphoribosylformylglycinamidine cyclo-ligase	0.91	*7.6E-01*	0.04	6.2E-07
BC0332	Phosphoribosylglycinamide formyltransferase	0.82	*3.7E-01*	0.05	1.6E-06
BC0333	IMP cyclohydrolase	0.90	*5.0E-01*	0.06	6.1E-06
BC0334	Phosphoribosylamine--glycine ligase	1.23	*6.6E-01*	0.22	9.2E-03
BC0344	Delta-1-pyrroline-5-carboxylate dehydrogenase	0.42	1.7E-03	0.42	8.4E-04
BC0366	hypothetical protein	1.09	*8.0E-01*	0.36	1.7E-04
BC0378	5-methylthioribose kinase	0.90	*6.8E-01*	0.46	1.0E-02
BC0410	Transcription regulator, Crp family	0.50	8.8E-03	0.36	1.7E-03
BC0422	Methyl-accepting chemotaxis protein	1.15	*5.6E-01*	0.34	2.3E-05
BC0423	Peptide synthetase	0.76	*3.8E-01*	0.45	3.7E-03
BC0442	Tellurium resistance protein terD	0.92	*5.8E-01*	4.88	3.0E-06
BC0443	Tellurium resistance protein terD	0.96	*8.2E-01*	3.95	3.0E-06
BC0444	Tellurium resistance protein terD	0.93	*7.0E-01*	3.41	2.9E-06
BC0450	Protein tyrosine phosphatase	0.85	*3.3E-01*	2.80	3.1E-05
BC0491	Formate acetyltransferase	0.10	1.2E-03	0.18	2.1E-04
BC0492	Pyruvate formate-lyase activating enzyme	0.07	2.8E-04	0.15	7.7E-04
BC0494	hypothetical Cytosolic Protein	0.54	*1.5E-01*	0.19	6.7E-06
BC0510	Integral membrane protein	1.87	3.2E-02	2.06	3.3E-04
BC0512	Glutamate-1-semialdehyde 2,1-aminomutase	1.00	*9.8E-01*	2.04	1.7E-04
BC0556	Microbial collagenase	0.50	4.3E-02	0.47	3.3E-02
BC0557	hypothetical protein	0.43	9.1E-03	0.33	4.9E-03
BC0558	Flottilin	0.57	*2.5E-01*	0.45	2.1E-02
BC0559	Methyl-accepting chemotaxis protein	0.71	*3.9E-01*	0.45	5.4E-03
BC0576	Methyl-accepting chemotaxis protein	0.49	*1.3E-01*	0.22	1.2E-03
BC0590	hypothetical protein	0.77	*1.2E-01*	0.49	6.8E-05
BC0598	Transcriptional activator NprR	0.45	1.3E-02	0.55	1.0E-03
BC0603	hypothetical Membrane Spanning Protein	1.43	*1.1E-01*	2.37	7.1E-04
BC0612	L-lactate permease	0.14	1.7E-02	0.54	1.9E-01
BC0615	Di-/tripeptide transporter	0.45	1.5E-02	0.54	1.2E-02
BC0619	Iron(III) dicitrate transport ATP-binding protein fecE	2.25	7.5E-03	0.86	6.7E-01
BC0621	2-amino-3-ketobutyrate coenzyme A ligase	0.68	*2.2E-01*	0.47	1.4E-03
BC0625	NorQ protein	1.34	*1.3E-01*	2.79	1.4E-04
BC0631	PTS system, trehalose-specific IIBC component	0.75	*2.0E-01*	0.24	3.0E-04
BC0632	Trehalose-6-phosphate hydrolase	0.81	*1.8E-01*	0.34	2.1E-04
BC0660	Ribokinase	0.86	*4.4E-01*	0.48	5.3E-03
BC0661	High affinity ribose transport protein rbsD	0.99	*9.5E-01*	0.44	1.5E-02
BC0668	(R,R)-butanediol dehydrogenase	0.42	2.1E-03	0.84	*3.5E-01*
BC0669	Potassium channel protein	0.74	*2.3E-01*	0.43	2.5E-04
BC0670	Phospholipase C	0.33	3.3E-02	0.24	4.6E-04
BC0671	Sphingomyelin phosphodiesterase	0.36	3.0E-02	0.34	9.8E-04
BC0695	Cytochrome aa3 quinol oxidase polypeptide IV	0.64	*5.6E-02*	0.42	2.3E-03
BC0696	Cytochrome aa3 quinol oxidase polypeptide III	0.50	1.7E-02	0.45	8.7E-04

BC0697	Cytochrome aa3 quinol oxidase polypeptide I	0.52	3.3E-02	0.43	2.7E-03
BC0698	Cytochrome aa3 quinol oxidase polypeptide II	0.51	*5.6E-02*	0.38	3.5E-05
BC0709	Ferrous iron transport protein A	0.38	9.6E-04	1.04	*9.5E-01*
BC0715	16S ribosomal RNA	1.34	*1.8E-01*	2.12	1.2E-02
BC0738	hypothetical protein	0.46	4.1E-03	0.66	*1.3E-01*
BC0753	Potassium-transporting ATPase A chain	0.39	1.0E-02	0.56	8.5E-03
BC0754	Potassium-transporting ATPase B chain	0.47	*5.0E-02*	0.44	1.2E-04
BC0786	Nitroreductase family	2.73	2.3E-02	1.38	*1.2E-01*
BC0805	outer surface protein	0.59	4.2E-02	0.29	4.8E-04
BC0806	Transcription antiterminator, BglG family	0.91	*6.0E-01*	0.44	8.7E-03
BC0813	enterotoxin / cell-wall binding protein entC	2.77	7.3E-04	6.35	6.3E-07
BC0814	ABC transporter permease protein	3.84	3.7E-03	2.67	1.8E-05
BC0815	ABC transporter ATP-binding protein	3.23	*8.6E-02*	4.47	1.6E-06
BC0816	periplasmic component of efflux system	3.27	*5.2E-02*	5.01	2.9E-07
BC0862	Protease I	1.15	*6.9E-01*	15.77	1.3E-05
BC0863	Catalase	1.01	*9.3E-01*	13.31	4.2E-06
BC0868	NADP-dependent glyceraldehyde-3-phosphate Dehydr.	1.09	*7.0E-01*	3.29	7.0E-06
BC0883	Acetolactate synthase large subunit	0.50	1.9E-02	2.36	6.3E-03
BC0884	Alpha-acetolactate decarboxylase	0.48	7.5E-03	1.62	6.5E-03
BC0887	Collagen adhesion protein	0.43	*1.0E-01*	0.32	2.6E-03
BC0888	N-acetylmuramoyl-L-alanine amidase	0.94	*8.1E-01*	0.35	7.0E-05
BC0892	hypothetical protein	2.03	3.8E-02	1.56	2.3E-02
BC0939	Type II restriction-modification system restriction SU	0.84	*5.3E-01*	0.48	5.1E-03
BC0964	Ribosomal-protein-alanine acetyltransferase	1.13	*4.4E-01*	2.42	2.6E-05
BC0998	General stress protein 17M	1.04	*8.7E-01*	11.41	2.1E-08
BC0999	hypothetical protein	1.07	*7.3E-01*	12.27	2.8E-07
BC1000	hypothetical Membrane Spanning Protein	0.83	*2.6E-01*	12.54	6.7E-06
BC1001	hypothetical protein	0.85	*5.6E-01*	2.02	6.7E-04
BC1002	Anti-sigma B factor antagonist	1.08	*6.8E-01*	5.36	2.5E-06
BC1003	Anti-sigma B factor	1.03	*8.6E-01*	8.97	1.4E-06
BC1004	RNA polymerase sigma-B factor	1.07	*6.4E-01*	7.84	1.8E-06
BC1010	hypothetical protein	0.97	*8.7E-01*	10.61	4.5E-06
BC1011	hypothetical protein	0.99	*9.7E-01*	4.31	2.7E-05
BC1012	hypothetical protein	1.06	*7.2E-01*	2.52	7.6E-04
BC1018	hypothetical protein	0.93	*7.4E-01*	2.28	7.3E-05
BC1049	Bis(5'-nucleosyl)-tetraphosphatase (asymmetrical)	0.80	*2.4E-01*	2.12	8.3E-05
BC1110	Cytotoxin K	0.47	1.4E-02	0.63	*1.8E-01*
BC1150	IG hypothetical 15940	0.76	*2.7E-01*	0.45	9.3E-04
BC1154	Ferrochelatase	0.39	2.6E-03	1.05	*9.4E-01*
BC1161	Peptidyl-prolyl cis-trans isomerase	1.17	*3.3E-01*	2.22	2.5E-04
BC1165	hydrolase (HAD superfamily)	0.93	*6.1E-01*	2.99	1.8E-05
BC1173	3-oxoacyl-[acyl-carrier-protein] synthase III	2.16	3.2E-03	1.17	*4.3E-01*
BC1176	hypothetical protein	2.39	6.4E-03	2.19	2.0E-03
BC1179	Oligopeptide-binding protein oppA	0.33	1.7E-03	1.82	*2.9E-01*
BC1180	Oligopeptide transport system permease protein oppB	0.33	5.6E-03	0.28	1.5E-04
BC1181	Oligopeptide transport system permease protein oppC	0.81	*5.5E-01*	0.22	8.2E-04

BC1182	Oligopeptide transport ATP-binding protein oppD	0.42	2.8E-02	0.28	6.5E-05
BC1183	Oligopeptide transport ATP-binding protein oppF	0.50	2.7E-02	0.45	5.3E-03
BC1185	Oligopeptide-binding protein oppA	0.58	3.5E-02	0.27	1.8E-03
BC1195	Globin Family Protein	1.11	*6.3E-01*	2.02	3.9E-03
BC1217	hypothetical protein	0.97	*8.7E-01*	0.32	1.8E-04
BC1270	hypothetical protein	0.43	3.9E-03	0.74	4.6E-02
BC1277	D-alanyl-D-alanine carboxypeptidase	0.84	*5.8E-01*	0.49	3.1E-03
BC1303	(S)-2-hydroxy-acid oxidase, iron-sulfur chain	0.24	2.2E-02	0.35	3.4E-03
BC1305	hypothetical protein	0.40	9.2E-03	0.49	1.0E-03
BC1307	hypothetical protein	0.74	*1.1E-01*	0.46	8.7E-04
BC1308	Nitrite transporter	0.35	2.9E-02	0.86	*7.9E-01*
BC1316	PhaR protein	0.42	8.8E-03	0.42	1.7E-04
BC1317	Acetoacetyl-CoA reductase	0.55	2.0E-02	0.44	7.4E-04
BC1318	Poly-beta-hydroxybutyrate polymerase	0.79	*3.6E-01*	0.46	4.2E-03
BC1346	hypothetical protein	0.94	*8.1E-01*	0.48	2.8E-02
BC1353	NrdI protein	1.96	1.1E-02	2.83	7.3E-05
BC1372	D-alanine-activating enzyme	1.34	*7.8E-02*	2.37	1.6E-04
BC1373	hypothetical protein	1.43	4.9E-02	2.97	6.7E-06
BC1376	Flavodoxin	2.17	1.6E-02	0.93	*9.0E-01*
BC1384	Bacitracin resistance protein	0.84	*5.2E-01*	0.40	8.4E-04
BC1420	hypothetical protein	0.99	*9.8E-01*	4.41	1.5E-05
BC1421	Phosphoadenosine phosphosulfate reductase	1.08	*6.9E-01*	3.07	3.5E-05
BC1422	Sulfate adenylyltransferase	1.08	*8.2E-01*	2.47	1.4E-04
BC1423	Adenylylsulfate kinase	0.97	*9.3E-01*	3.44	2.1E-04
BC1424	Ferredoxin--nitrite reductase	ND	ND	3.19	4.2E-03
BC1425	hypothetical protein	0.97	*8.5E-01*	2.36	3.5E-03
BC1432	Proton/sodium-glutamate symport protein	0.77	*4.2E-01*	0.45	7.1E-04
BC1435	hypothetical protein	14.02	2.3E-02	33.96	2.1E-08
BC1436	Phage shock protein A	11.94	2.2E-02	12.83	7.9E-07
BC1437	Transporter yvqF	3.47	4.3E-03	2.19	6.9E-03
BC1438	Two-component sensor protein yvqE	2.29	2.1E-02	1.75	2.8E-02
BC1439	Two-component response regulator yvqC	2.31	1.2E-02	2.18	2.6E-03
BC1448	Nitric oxide dioxygenase	0.47	2.3E-03	0.50	2.8E-02
BC1461	DNA integration/recombination/invertion protein	1.59	2.3E-02	4.06	1.7E-05
BC1528	hypothetical Membrane Spanning Protein	1.00	*1.0E+00*	2.23	1.7E-04
BC1531	hypothetical Transcriptional Regulatory Protein	1.20	*2.6E-01*	2.09	7.7E-04
BC1540	3-methyl-2-oxobutanoate hydroxymethyltransferase	1.63	2.3E-02	0.38	6.6E-04
BC1541	Pantoate--beta-alanine ligase	1.42	*6.4E-02*	0.41	1.1E-03
BC1542	Aspartate 1-decarboxylase	1.26	*1.5E-01*	0.48	1.2E-03
BC1553	Multimodular transpeptidase-transglycosylase	2.08	4.2E-03	1.25	*1.2E-01*
BC1569	Xanthine phosphoribosyltransferase	2.13	1.7E-02	0.21	4.7E-04
BC1602	hypothetical Cytosolic Protein	0.65	*7.7E-02*	0.33	7.0E-04
BC1625	Chemotaxis motA protein	0.81	*2.6E-01*	0.40	7.1E-04
BC1626	Chemotaxis motB protein	0.92	*7.8E-01*	0.39	2.1E-03
BC1628	Chemotaxis protein cheA	1.04	*8.7E-01*	0.47	4.2E-03
BC1629	Chemotaxis protein cheC	0.89	*4.3E-01*	0.46	2.6E-03

BC1630	hypothetical protein	1.01	*9.8E-01*	0.38	7.3E-04
BC1634	UDP-N-acetylenolpyruvoylglucosamine reductase	0.83	*2.6E-01*	0.47	2.4E-03
BC1636	Flagellar hook-associated protein 1	1.07	*7.2E-01*	0.47	3.4E-05
BC1637	Flagellar hook-associated protein 3	0.94	*7.7E-01*	0.41	3.1E-05
BC1640	hypothetical protein	0.90	*5.6E-01*	0.41	5.7E-04
BC1641	Flagellar basal-body rod protein flgB	0.95	*8.2E-01*	0.45	1.7E-03
BC1642	Flagellar basal-body rod protein flgC	1.10	*6.2E-01*	0.44	3.5E-05
BC1643	Flagellar hook-basal body complex protein fliE	1.06	*8.6E-01*	0.37	8.0E-04
BC1644	Flagellar M-ring protein fliF	0.96	*8.5E-01*	0.31	1.8E-05
BC1645	Flagellar motor switch protein fliG	1.15	*5.7E-01*	0.35	3.1E-04
BC1646	hypothetical protein	1.13	*6.1E-01*	0.48	2.3E-03
BC1649	similar to hypothetical protein	0.84	*3.5E-01*	0.47	7.1E-04
BC1650	Basal-body rod modification protein flgD	0.88	*4.4E-01*	0.44	3.1E-05
BC1651	Flagellar hook protein flgE	0.89	*5.0E-01*	0.41	1.2E-04
BC1652	hypothetical protein	0.99	*9.4E-01*	0.38	4.6E-04
BC1653	hypothetical protein	0.91	*6.7E-01*	0.45	4.1E-05
BC1654	Chemotaxis protein cheV	0.92	*7.3E-01*	0.40	4.5E-05
BC1656	Flagellin	0.77	*1.3E-01*	0.40	2.7E-05
BC1657	Flagellin	0.57	*5.0E-02*	0.18	1.8E-06
BC1658	Flagellin	0.55	6.3E-03	0.25	4.6E-04
BC1659	Flagellin	0.55	3.0E-02	0.19	6.7E-05
BC1663	Flagellar motor switch protein fliN	1.01	*9.5E-01*	0.47	1.2E-03
BC1664	Flagellar motor switch protein fliN	0.91	*6.3E-01*	0.43	2.8E-03
BC1666	Flagellar biosynthetic protein fliQ	1.07	*7.6E-01*	0.46	1.8E-04
BC1667	Flagellar biosynthetic protein fliR	0.97	*8.6E-01*	0.50	5.1E-03
BC1668	Flagellar biosynthetic protein flhB	1.22	*4.5E-01*	0.47	1.9E-03
BC1669	similar to Flagellar biosynthesis protein flhA	1.01	*9.7E-01*	0.35	1.8E-05
BC1670	similar to Flagellar biosynthesis protein flhF	1.12	*6.9E-01*	0.44	1.4E-03
BC1760	3-oxoacyl-[acyl-carrier-protein] synthase III	2.90	1.1E-02	5.06	2.6E-06
BC1774	Peptide methionine sulfoxide reductase	0.90	*5.6E-01*	2.56	6.9E-05
BC1804	Rhodanese-related sulfurtransferases	3.84	2.9E-03	1.87	1.1E-03
BC1851	Transcriptional regulator	0.87	*6.4E-01*	0.29	1.8E-03
BC1852	Exonuclease SbcC	0.69	*9.1E-02*	0.20	3.4E-04
BC1853	hypothetical protein	0.74	*3.7E-01*	0.23	4.0E-04
BC1854	hypothetical Cytosolic Protein	0.78	*3.4E-01*	0.20	1.4E-04
BC1855	Chromosome segregation ATPases	0.99	*9.3E-01*	0.22	8.2E-04
BC1856	Phage protein	1.24	*5.4E-01*	0.26	7.1E-04
BC1857	Exonuclease SbcD	0.75	*2.9E-01*	0.20	4.5E-04
BC1858	Phage protein	0.99	*9.7E-01*	0.27	2.4E-03
BC1859	Phage protein	ND	ND	0.43	6.9E-03
BC1860	Phage protein	0.99	*9.6E-01*	0.30	3.3E-03
BC1861	DNA/RNA helicase (DEAD/DEAH box family)	0.84	*4.1E-01*	0.20	3.2E-05
BC1862	Phage protein	ND	ND	0.41	1.5E-02
BC1863	MCM domain family protein	1.09	*7.9E-01*	0.42	9.8E-03
BC1864	DNA polymerase I	1.12	*5.6E-01*	0.46	4.6E-03
BC1865	Phage protein	ND	ND	0.36	2.7E-03

BC1866	Phage protein	1.09	*8.0E-01*	0.40	8.5E-03
BC1867	Phage protein	ND	ND	0.46	1.5E-03
BC1868	Phage protein	1.33	*5.6E-01*	0.46	1.7E-02
BC1869	Phage protein	1.11	*7.5E-01*	0.48	5.1E-03
BC1871	Phage protein	1.16	*6.8E-01*	0.39	1.5E-03
BC1872	Phage protein	0.91	*7.1E-01*	0.39	5.1E-03
BC1873	Phage protein	1.00	*9.9E-01*	0.46	5.4E-03
BC1875	Phage protein	1.07	*9.0E-01*	0.44	3.7E-03
BC1876	Phage protein	0.97	*8.9E-01*	0.48	4.0E-03
BC1881	Phage protein	1.13	*6.0E-01*	0.48	2.1E-02
BC1882	Phage protein	0.92	*7.1E-01*	0.44	3.4E-03
BC1883	Phage protein	1.01	*9.5E-01*	0.45	1.9E-03
BC1938	Cytochrome d ubiquinol oxidase subunit I	1.48	*1.2E-01*	2.59	4.7E-04
BC1939	Cytochrome d ubiquinol oxidase subunit II	1.28	*4.8E-01*	6.31	2.3E-05
BC1940	Transport ATP-binding protein cydD	1.00	*9.9E-01*	3.12	7.1E-04
BC1941	Transport ATP-binding protein cydC	1.31	*4.0E-01*	2.24	8.2E-04
BC1974	Peptidoglycan N-acetylglucosamine deacetylase	0.82	*6.2E-01*	0.43	1.0E-02
BC1991	putative murein endopeptidase	1.24	*2.7E-01*	4.89	1.9E-06
BC1995	ABC transporter permease protein	3.52	7.7E-03	2.67	8.1E-05
BC2056	hypothetical protein	0.35	5.0E-03	0.16	3.4E-07
BC2057	Stomatin like protein	0.41	2.2E-03	0.30	5.5E-04
BC2118	Respiratory nitrate reductase alpha chain	0.39	1.3E-03	0.22	1.4E-03
BC2119	Respiratory nitrate reductase beta chain	0.46	2.1E-02	0.07	2.2E-04
BC2120	Respiratory nitrate reductase delta chain	0.87	*6.7E-01*	0.20	3.2E-02
BC2121	Respiratory nitrate reductase gamma chain	0.90	*6.2E-01*	0.27	2.4E-02
BC2123	Molybdenum cofactor biosynthesis protein A	0.14	3.1E-03	0.69	*4.0E-01*
BC2124	Molybdopterin biosynthesis MoeB protein	0.35	1.3E-02	0.54	4.7E-02
BC2125	Molybdopterin biosynthesis MoeA protein	0.54	*1.2E-01*	0.48	2.5E-03
BC2126	Molybdopterin (MPT) converting factor, subunit 2	ND	ND	0.21	1.1E-02
BC2128	Nitrite extrusion protein	1.00	*9.9E-01*	0.21	1.8E-02
BC2132	Ferrochelatase	0.88	*5.3E-01*	0.36	4.2E-02
BC2133	CbiX protein	0.49	*2.0E-01*	0.21	6.3E-03
BC2134	Uroporphyrin-III C-methyltransferase	0.86	*7.6E-01*	0.29	3.7E-03
BC2135	Nitrite reductase [NAD(P)H] small subunit	0.40	1.5E-02	0.34	6.0E-03
BC2136	Nitrite reductase [NAD(P)H] large subunit	0.46	5.3E-03	0.50	6.3E-03
BC2152	hypothetical protein	0.92	*6.4E-01*	0.49	8.6E-04
BC2193	MecA protein	0.68	*3.3E-01*	0.28	5.5E-04
BC2220	Alcohol dehydrogenase	0.28	5.4E-04	0.56	9.7E-03
BC2223	Gluconokinase	0.78	*2.8E-01*	0.30	5.0E-04
BC2224	Gluconate permease	0.86	*7.7E-01*	0.30	1.7E-04
BC2225	6-phosphogluconate dehydrogenase	0.70	*2.5E-01*	0.34	2.8E-04
BC2268	hypothetical protein	1.14	*5.2E-01*	2.87	7.1E-05
BC2272	Protein export protein prsA precursor	1.91	3.6E-02	3.33	7.6E-06
BC2303	Isochorismate synthase	3.08	2.6E-03	1.02	*9.9E-01*
BC2304	2,3-dihydroxybenzoate-AMP ligase	3.40	5.5E-03	1.00	*9.9E-01*
BC2305	Isochorismatase	3.46	8.9E-03	0.98	*9.9E-01*

BC2306	Glycine-AMP ligase	4.80	2.5E-03	0.88	*6.7E-01*
BC2307	Glycine-AMP ligase	3.51	1.2E-03	0.80	*4.1E-01*
BC2308	Glycine-AMP ligase	7.89	6.6E-04	1.07	*9.1E-01*
BC2309	Antibiotic/siderophore biosynthesis protein	2.90	8.1E-03	1.20	*8.3E-01*
BC2310	Multidrug resistance protein B	3.06	2.4E-03	0.88	*8.1E-01*
BC2312	hypothetical protein	2.89	2.7E-03	0.97	*9.8E-01*
BC2343	Transposase	2.77	3.8E-02	3.17	2.6E-05
BC2390	hypothetical protein	3.36	2.0E-02	2.95	3.1E-05
BC2461	Antigen	2.50	9.2E-03	3.86	7.8E-06
BC2552	hypothetical protein	0.79	*1.3E-01*	0.39	6.7E-05
BC2603	hypothetical protein	0.97	*9.4E-01*	2.29	2.2E-04
BC2620	Penicillin-binding protein transpeptidase	2.49	2.7E-02	4.95	1.6E-07
BC2705	hypothetical Cytosolic Protein	1.04	*7.9E-01*	2.02	2.2E-03
BC2750	hypothetical protein	0.83	*5.4E-01*	0.48	1.5E-02
BC2841	hypothetical protein	ND	ND	0.48	9.4E-05
BC2842	hypothetical protein	0.90	*6.0E-01*	0.45	1.3E-03
BC2895	hypothetical protein	3.05	9.1E-03	4.54	1.3E-06
BC2952	enterotoxin / cell-wall binding protein entB	0.71	*2.9E-01*	0.29	1.7E-03
BC2984	Immune inhibitor A precursor	4.05	2.4E-02	9.79	2.9E-07
BC2985	Vancomycin B-type resistance protein vanW	2.82	*8.5E-02*	8.63	1.6E-06
BC3000	Proline/betaine transporter	1.56	2.8E-02	2.48	1.5E-03
BC3021	hypothetical Cytosolic Protein	0.69	*1.1E-01*	0.37	4.7E-04
BC3022	hypothetical protein	1.02	*9.5E-01*	0.34	6.3E-04
BC3069	Transcriptional regulator kipR	0.87	*6.1E-01*	0.48	2.9E-02
BC3093	Aspartate ammonia-lyase	2.13	1.3E-02	1.15	*5.0E-01*
BC3094	L-asparaginase	2.81	3.7E-03	1.04	*9.2E-01*
BC3117	Arsenical pump membrane protein	2.12	2.8E-02	1.81	1.8E-03
BC3121	5'-nucleotidase	0.79	*1.5E-01*	0.44	9.3E-04
BC3129	Magnesium and cobalt transport protein corA	0.84	*5.9E-01*	2.65	5.6E-05
BC3130	hypothetical protein	1.14	*6.7E-01*	5.30	7.4E-05
BC3131	hypothetical protein	0.87	*7.6E-01*	2.40	5.5E-04
BC3132	General stress protein 17M	ND	ND	4.06	1.3E-05
BC3249	Phosphoserine aminotransferase	0.93	*7.4E-01*	2.11	7.2E-05
BC3328	ABC transporter permease protein	0.71	*7.1E-02*	0.41	3.6E-03
BC3351	hypothetical protein	0.89	*5.3E-01*	0.38	1.2E-04
BC3380	Quinone oxidoreductase	1.15	*3.6E-01*	2.04	6.6E-05
BC3461	LMBE-related protein	1.21	*3.5E-01*	2.12	3.3E-04
BC3462	hypothetical protein	1.19	*3.3E-01*	2.67	3.6E-05
BC3466	Ferrichrome-binding protein	2.34	7.9E-03	2.19	*3.0E-01*
BC3467	Ferrichrome transport system permease protein fhuG	2.71	7.0E-03	1.11	*9.0E-01*
BC3468	Ferrichrome transport system permease protein fhuB	2.31	3.1E-02	1.35	*1.6E-01*
BC3540	BNR-repeat containing protein	2.95	2.8E-03	1.19	*5.8E-01*
BC3541	Flavodoxin	2.04	3.2E-02	1.26	*6.8E-01*
BC3555	Aldehyde dehydrogenase	1.03	*8.4E-01*	2.02	9.5E-05
BC3586	Oligopeptide-binding protein oppA	0.38	1.1E-03	0.18	2.7E-03
BC3600	Protease HhoA	1.35	*1.5E-01*	2.10	6.3E-04

BC3625	Phosphohydrolase (MutT/nudix family protein)	0.94	*8.6E-01*	0.35	2.3E-02
BC3663	Transporter, Drug/Metabolite Exporter family	1.40	*1.3E-01*	2.06	7.9E-04
BC3680	hypothetical Exported Protein	0.91	*6.4E-01*	0.29	9.4E-07
BC3681	IG hypothetical 18106	0.92	*6.0E-01*	0.31	2.1E-04
BC3682	Transketolase	0.87	*5.0E-01*	0.45	1.2E-04
BC3705	similar to Glutamine synthetase; EC_number 6.3.1.2	2.27	2.1E-03	0.56	3.8E-03
BC3706	Transcriptional regulator, MerR family	2.52	4.6E-04	1.17	*3.2E-01*
BC3722	hypothetical Membrane Spanning Protein	0.74	*2.3E-01*	0.43	1.6E-03
BC3723	hypothetical protein	0.98	*8.8E-01*	2.43	1.9E-05
BC3736	Iron(III) dicitrate transport system permease protein	2.16	5.8E-03	1.25	*6.1E-01*
BC3738	Iron(III) dicitrate-binding protein	2.07	1.3E-02	1.22	*6.5E-01*
BC3777	IG hypothetical 15594	0.86	*5.4E-01*	0.42	1.2E-03
BC3786	Zinc protease	1.17	*3.3E-01*	2.16	1.1E-04
BC3787	Zinc protease	1.10	*5.9E-01*	2.55	1.5E-05
BC3788	Nucleoside transport system permease protein	0.99	*9.5E-01*	0.06	2.9E-07
BC3790	Nucleoside transport ATP-binding protein	1.03	*9.3E-01*	0.11	3.5E-05
BC3791	Nucleoside-binding protein	1.26	*3.2E-01*	0.06	1.7E-06
BC3792	Transcriptional regulator, GntR family	1.99	2.2E-02	0.09	1.4E-05
BC3804	Chitooligosaccharide deacetylase	1.05	*8.1E-01*	0.39	1.6E-03
BC3817	Prolyl-tRNA synthetase	0.96	*8.6E-01*	0.49	4.0E-03
BC3818	similar to membrane metalloprotease	0.87	*5.0E-01*	0.35	1.5E-05
BC3819	1-deoxy-D-xylulose 5-phosphate reductoisomerase	0.87	*4.6E-01*	0.47	1.1E-04
BC3820	Phosphatidate cytidylyltransferase	0.92	*7.3E-01*	0.30	6.6E-06
BC3821	Undecaprenyl pyrophosphate synthetase	0.88	*5.7E-01*	0.31	1.7E-05
BC3822	Ribosome Recycling Factor (RRF)	0.92	*6.0E-01*	0.38	5.6E-06
BC3823	Uridylate kinase	1.02	*8.9E-01*	0.36	1.5E-05
BC3824	Protein Translation Elongation Factor Ts (EF-Ts)	1.05	*7.6E-01*	0.45	2.4E-05
BC3841	RNA binding protein	1.09	*6.4E-01*	0.43	2.8E-03
BC3842	SSU ribosomal protein S16P	1.04	*7.8E-01*	0.50	1.5E-04
BC3881	Phosphoglycolate phosphatase	0.85	*3.7E-01*	0.48	1.2E-03
BC3882	Orotate phosphoribosyltransferase	0.99	*9.7E-01*	0.36	3.1E-02
BC3883	Orotidine 5'-phosphate decarboxylase	1.08	*8.3E-01*	0.32	4.8E-02
BC3884	Dihydroorotate dehydrogenase, catalytic subunit	0.89	*7.5E-01*	0.25	5.8E-03
BC3885	Dihydroorotate dehydrogenase electron transfer SU	0.76	*4.4E-01*	0.28	1.4E-02
BC3887	Carbamoyl-phosphate synthase small chain	0.57	*3.1E-01*	0.24	3.5E-04
BC3888	Dihydroorotase	0.57	*3.4E-01*	0.26	4.7E-04
BC3894	DnaK suppressor protein	1.06	*8.7E-01*	0.34	1.8E-03
BC3943	Cytochrome c oxidase polypeptide I	1.15	*4.8E-01*	0.46	6.4E-03
BC3944	Cytochrome c oxidase polypeptide II	1.25	*2.9E-01*	0.49	3.8E-03
BC3981	Tetrahydrodipicolinate N-acetyltransferase	1.16	*4.0E-01*	2.29	2.4E-05
BC4003	5-MTHT homocysteine methyltransferase	0.42	*1.2E-01*	2.37	3.0E-04
BC4032	Methylthioribose salvage protein	1.10	*5.5E-01*	2.85	1.7E-05
BC4033	5-methylthioribose kinase	1.03	*9.2E-01*	2.37	7.0E-05
BC4036	Ribulose bisphosphate carboxylase large chain	0.97	*9.0E-01*	2.89	1.2E-05
BC4037	Methylthioribose salvage protein	0.99	*9.8E-01*	2.39	1.4E-04
BC4038	Methylthioribose salvage protein	1.00	*9.9E-01*	2.11	3.6E-05

BC4091	Ferric uptake regulation protein	0.95	*7.3E-01*	2.15	6.1E-04
BC4122	similar to 2',3'-cyclic-nucleotide 2'-phosphodiesterase	0.71	*1.7E-01*	0.27	2.7E-05
BC4148	Arginine transport ATP-binding protein artP	0.88	*7.3E-01*	0.36	2.5E-02
BC4149	Arginine transport system permease protein artQ	0.59	4.4E-02	0.42	2.3E-02
BC4150	Arginine-binding protein	0.29	4.3E-03	0.21	9.3E-03
BC4162	Leucine dehydrogenase	1.09	*5.6E-01*	2.43	2.1E-04
BC4224	Glycine dehydrogenase [decarboxylating]	0.64	*5.2E-02*	0.43	1.3E-04
BC4225	Glycine dehydrogenase [decarboxylating]	0.61	*6.4E-02*	0.47	7.0E-05
BC4240	Transcriptional regulator	0.28	8.2E-03	0.43	6.3E-03
BC4253	Cystathionine gamma-synthase	1.03	*9.5E-01*	2.05	3.1E-04
BC4263	LSU ribosomal protein L33P	0.70	*1.5E-01*	0.34	1.7E-04
BC4271	Transporter, MFS superfamily	0.91	*7.4E-01*	0.37	4.5E-04
BC4290	DNA primase	1.03	*8.7E-01*	2.18	1.5E-04
BC4320	SSU ribosomal protein S20P	0.66	*7.1E-02*	0.30	4.2E-06
BC4341	GTP pyrophosphokinase	0.98	*9.0E-01*	3.27	2.9E-05
BC4365	Alcohol dehydrogenase	0.11	1.5E-04	0.83	*6.3E-01*
BC4366	Cystathionine beta-lyase	1.18	*5.4E-01*	2.64	1.7E-03
BC4367	Cysteine synthase	1.26	*5.1E-01*	2.27	8.5E-03
BC4368	S-Adenosylhomocysteine nucleosidase	1.09	*7.4E-01*	3.08	1.4E-03
BC4369	Dimethyladenosine transferase	0.94	*8.8E-01*	2.84	8.4E-04
BC4373	hypothetical protein	0.94	*6.9E-01*	0.43	9.3E-04
**BC4405**	**Protein translocase subunit SecD / SecF**	**0.26**	***5.8E-02***	**0.13**	**1.0E-05**
BC4427	Prephenate dehydratase	0.77	*1.2E-01*	2.01	5.2E-05
BC4432	Two-component sensor kinase yvcQ	0.90	*7.5E-01*	0.49	4.4E-04
BC4446	Rod shape-determining protein mreB	2.10	2.4E-03	1.43	3.5E-02
BC4452	hypothetical protein	ND	ND	0.39	1.1E-02
BC4482	hypothetical protein	6.28	9.0E-04	5.32	6.5E-05
BC4486	hypothetical protein	0.83	*5.3E-01*	0.37	1.1E-04
BC4487	Superfamily I DNA and RNA helicases	0.80	*3.9E-01*	0.33	3.4E-04
BC4512	Chemotaxis motB protein	0.69	4.6E-02	0.29	5.5E-05
BC4513	Chemotaxis motA protein	0.65	*7.6E-02*	0.28	2.7E-05
BC4516	Succinate dehydrogenase iron-sulfur protein	0.99	*9.7E-01*	0.49	3.4E-03
BC4523	Electron transfer flavoprotein beta-subunit	ND	ND	0.48	1.3E-02
BC4548	Cell surface protein	1.20	*5.1E-01*	3.12	3.2E-02
BC4564	Metal-dependent phosphohydrolase	1.13	*5.7E-01*	2.11	1.5E-04
BC4582	S-adenosylmethionine decarboxylase proenzyme	1.31	*1.7E-01*	2.62	4.2E-05
BC4610	hypothetical protein	0.75	*2.8E-01*	0.35	8.2E-04
BC4614	Xaa-Pro dipeptidase	0.70	*8.2E-02*	0.47	6.1E-05
BC4625	Universal stress protein family	0.35	3.9E-02	0.47	6.7E-03
BC4630	Argininosuccinate synthase	0.50	4.4E-03	0.49	8.6E-03
BC4639	Thiol peroxidase	0.68	*1.3E-01*	0.48	3.2E-04
BC4692	hypothetical protein	0.76	*1.1E-01*	0.41	1.7E-04
BC4703	Transcriptional regulator, DeoR family	0.68	2.9E-02	2.68	7.0E-05
BC4742	ABC transporter permease protein	3.54	5.5E-03	3.36	3.4E-05
BC4743	ABC transporter ATP-binding protein	3.22	1.5E-02	4.72	2.3E-07
BC4752	hypothetical protein	1.34	*2.6E-01*	2.53	3.4E-05

BC4775	Phosphoglycerol transferase	1.27	*2.3E-01*	2.06	4.2E-04
BC4789	Autoinducer-2 production protein luxS	0.74	*4.9E-01*	2.12	4.5E-03
BC4792	Cytochrome d ubiquinol oxidase subunit I	0.15	9.7E-04	0.14	8.6E-05
BC4793	Cytochrome d ubiquinol oxidase subunit II	0.14	2.1E-04	0.11	8.1E-04
BC4802	hypothetical protein	1.96	*1.2E-01*	3.05	4.8E-05
BC4813	hypothetical protein	2.94	*6.8E-02*	14.25	1.8E-07
BC4817	hypothetical protein	1.44	*2.0E-01*	2.03	8.7E-04
BC4830	ABC transporter permease protein	2.40	3.5E-03	2.86	1.1E-05
BC4831	ABC transporter ATP-binding protein	2.92	2.0E-02	6.68	3.9E-08
BC4870	L-lactate dehydrogenase	0.12	2.4E-02	0.50	*2.3E-01*
BC4880	tRNA-Met	1.07	*7.8E-01*	0.47	6.1E-04
BC4897	IG hypothetical 16740	0.79	*5.0E-01*	0.42	6.3E-03
BC4915	ComA operon protein 2	1.09	*6.1E-01*	2.51	6.8E-05
BC4927	Cell surface protein	0.82	*2.7E-01*	0.29	3.1E-04
BC4995	regulatory protein (pfoS/R)	0.36	3.1E-03	0.25	3.2E-03
BC5006	Prolyne dehydrogenase	0.42	7.6E-03	0.32	3.1E-03
BC5034	Methyl-accepting chemotaxis protein	0.88	*7.4E-01*	0.39	6.3E-03
BC5044	Non-specific DNA-binding protein Dps / Ferroxidase	0.44	3.0E-03	0.61	3.9E-03
BC5051	Sodium/proton-dependent alanine carrier protein	2.49	1.4E-03	1.17	*5.2E-01*
BC5085	hypothetical Cytosolic Protein	1.02	*9.4E-01*	0.43	8.2E-03
BC5086	putative lantibiotic biosynthesis protein	0.81	*3.8E-01*	0.39	5.4E-03
BC5106	Ferric anguibactin-binding protein	2.02	1.1E-02	1.13	*8.2E-01*
BC5116	hypothetical protein	0.59	*1.1E-01*	0.16	1.3E-05
BC5117	ABC transporter permease protein	0.54	*1.2E-01*	0.11	1.6E-06
BC5118	ABC transporter ATP-binding protein	0.69	*3.1E-01*	0.12	3.1E-05
BC5119	hypothetical protein	0.63	*2.4E-01*	0.12	2.8E-05
BC5120	hypothetical Cytosolic Protein	0.69	*2.6E-01*	0.12	6.7E-06
BC5121	hypothetical protein	0.73	*2.7E-01*	0.12	1.7E-05
BC5122	hypothetical Cytosolic Protein	0.54	*6.2E-02*	0.18	2.4E-05
BC5123	hypothetical protein	0.93	*8.0E-01*	0.16	3.6E-05
BC5124	hypothetical protein	0.74	*2.6E-01*	0.19	2.7E-05
BC5125	hypothetical protein	0.71	*1.1E-01*	0.33	1.5E-05
BC5138	similar to Phosphoglycerate kinase	0.63	*8.8E-02*	2.02	2.8E-03
BC5141	Central glycolytic genes regulator	0.61	1.9E-02	2.20	2.7E-04
BC5159	Thioredoxin reductase	1.19	*2.6E-01*	2.01	3.7E-04
BC5179	Transposase	3.28	3.7E-02	3.79	1.5E-06
BC5180	Transposase	2.96	2.2E-02	3.28	8.8E-06
BC5190	Probable Sigma (54) modulation protein	0.36	8.3E-04	2.49	1.4E-04
BC5191	Cold shock protein	2.02	1.1E-02	1.11	*7.1E-01*
BC5203	Undecaprenyl-P α-N-acetylglucosamine-PT	1.58	1.7E-02	2.00	1.7E-04
BC5219	Integral membrane protein	0.56	*5.9E-02*	0.22	1.1E-04
BC5228	L-lactate permease	1.16	*5.3E-01*	0.28	2.4E-03
BC5233	D-alanine aminotransferase	0.89	*4.7E-01*	2.41	2.8E-05
BC5239	enterotoxin / cell-wall binding protein entA	3.88	2.6E-03	5.60	7.9E-07
BC5241	IG hypothetical 16680	0.21	3.2E-02	0.37	2.3E-04
BC5242	Membrane protein with C2C2 zinc finger	0.62	*5.1E-01*	0.23	1.1E-04

BC5243	hypothetical protein	0.68	*4.7E-01*	0.20	9.1E-05
BC5252	hypothetical Membrane Spanning Protein	0.50	*8.5E-02*	0.11	2.3E-06
BC5253	ABC transporter permease protein	0.44	*6.5E-02*	0.08	9.1E-06
BC5254	ABC transporter ATP-binding protein	0.54	*1.0E-01*	0.11	5.0E-06
BC5255	periplasmic component of efflux system	0.46	*9.2E-02*	0.08	8.2E-07
BC5264	EPSX protein	1.10	*8.1E-01*	0.50	1.1E-03
BC5266	Heteropolysaccharide repeat unit export protein	0.91	*7.4E-01*	0.46	1.5E-04
BC5270	Undecaprenyl-phosphate galactosephosphotransferase	0.52	*1.4E-01*	0.32	1.4E-04
BC5271	UDP-N-acetylglucosamine 4-epimerase	0.98	*9.4E-01*	0.36	3.3E-04
BC5272	Carbamoyl-phosphate synthase small chain	0.70	*2.1E-01*	0.35	2.3E-04
BC5273	UDP-bacillosamine synthetase	1.00	*9.9E-01*	0.29	7.8E-06
BC5274	UDP-N-acetylglucosamine 4,6-dehydratase	1.09	*8.0E-01*	0.28	1.5E-04
BC5275	UTP-glucose-1-phosphate uridylyltransferase	1.02	*9.3E-01*	0.42	5.1E-03
BC5277	Tyrosine-protein kinase	1.34	*4.3E-01*	0.37	1.5E-04
BC5278	Chain length regulator	1.31	*3.6E-01*	0.39	2.2E-04
BC5285	Bacitracin transport ATP-binding protein bcrA	0.93	*7.7E-01*	0.34	5.0E-03
BC5287	Stage II sporulation protein D	0.58	*1.8E-01*	0.43	5.8E-04
BC5303	Integral membrane protein	0.84	*4.3E-01*	0.49	4.9E-03
BC5305	ATP synthase epsilon chain	0.54	5.4E-03	0.31	6.6E-05
BC5306	ATP synthase beta chain	0.56	1.4E-02	0.30	1.4E-04
BC5307	ATP synthase gamma chain	0.54	1.5E-02	0.29	4.9E-04
BC5308	ATP synthase alpha chain	0.55	1.7E-02	0.30	2.5E-04
BC5309	ATP synthase delta chain	0.57	2.2E-02	0.24	7.3E-05
BC5310	ATP synthase B chain	0.55	1.9E-02	0.25	1.2E-04
BC5311	ATP synthase C chain	0.60	1.3E-02	0.33	7.0E-05
BC5312	ATP synthase A chain	0.62	2.2E-02	0.34	1.2E-04
BC5315	Uracil phosphoribosyltransferase	0.85	*3.5E-01*	0.47	6.4E-05
BC5316	Serine hydroxymethyltransferase	0.81	*2.8E-01*	0.24	9.1E-04
BC5333	Fructose-1,6-bisphosphatase	0.63	1.9E-02	0.41	1.6E-04
BC5338	CTP synthase	0.90	*6.8E-01*	0.44	9.4E-04
BC5351	Bacillolysin	0.83	*2.4E-01*	4.16	5.7E-06
BC5357	Collagen adhesion protein	0.68	*2.5E-01*	0.24	5.4E-05
BC5361	ECF-type sigma factor negative effector	3.42	4.1E-02	12.40	1.7E-06
BC5362	ECF-type sigma factor negative effector	2.97	1.9E-02	8.26	2.4E-08
BC5363	RNA polymerase ECF-type sigma factor	2.60	*1.1E-01*	16.82	4.8E-07
BC5369	hypothetical protein	0.88	*4.3E-01*	0.47	6.1E-03
BC5380	Ferrichrome-binding protein	1.28	*2.9E-01*	0.30	1.7E-04
BC5382	Ferrichrome transport system permease protein fhuG	2.36	6.3E-03	0.89	*6.0E-01*
BC5383	Ferrichrome transport system permease protein fhuB	2.20	1.0E-02	1.16	*8.1E-01*
BC5397	hypothetical Membrane Spanning Protein	1.98	3.8E-02	2.38	4.9E-04
BC5406	O-acetylhomoserine sulfhydrylase	1.20	*7.1E-01*	2.04	1.3E-03
BC5422	hypothetical protein	2.02	1.1E-02	0.72	4.7E-02
BC5428	hypothetical protein	0.85	*6.4E-01*	0.37	1.4E-04
BC5439	Murein hydrolase exporter	1.79	4.9E-02	0.40	3.3E-03
BC5445	Superoxide dismutase [Mn]	0.43	1.5E-03	0.61	3.5E-03
BC5452	GMP reductase	1.25	*5.2E-01*	0.30	6.6E-04

BC5466	tRNA-Glu	1.02	*9.0E-01*	0.42	1.1E-03
BC5473	IG hypothetical 15508	0.86	*6.1E-01*	0.33	8.3E-05

The list shows at least 2-fold differentially regulated genes in the *B. cereus* ATCC 1459 Δ*secDF* mutant compared to wild type (P-value < 0.05).

[1] FC -fold change of transcriptional expression Δ*secDF* mutant/wild type determined by Bayesian linear modelling using the Limma-package.

[2] uncorrected P-values; in *italic* > 0.05; two independent cultures

[3] P-values were computed using false discovery rate correction of 0.05; six independent cultures

ND no data
