# Supplementary material for: SecDF as Part of the Sec-Translocase Facilitates Efficient Secretion of Bacillus cereus Toxins and Cell Wall-Associated Proteins
Source: PLoS One. 2014 Aug 1;9(8):e103326. doi: 10.1371/journal.pone.0103326 (PMC4118872; doi:10.1371/journal.pone.0103326)
Supplement: Supplemental information S1 — Materials and methods. (PDF) [file pone.0103326.s011.pdf]

Supplemental information S1: Materials and methods

Oligonucleotides, plasmids and strains used in this study

**Oligonucleotides**	**gene**	**Sequence 5´- 3´, restriction sides are underlined**	
Construction of the secDF markerless mutant			
Bc4405_up_XbaI_F	*secDF*	TGATCTAGACCGCGGACAGTTACAAGTCG	
Bc4405_up_SalI_R	*secDF*	ATAGTCGACCATATGTACCCTCTTTCTGCTACTAAC	
Bc4405_down_SalI_F	*secDF*	ACAGTCGACTAGAAAAGATGA TGTCTCTTTATGAGG	
Bc4405_down_PstI_R	*secDF*	ATACTGCAGTCGGCGGTTCTAGTTCTTGC	
sequBc4405_F	*secDF*	ATGCGGTTGATAAAGCCGAG	
sequBc4405_R	*secDF*	CATCCGAACGGTGCTCATATGC	
Cloning of secDF into expression vectors			*vector*
Bc4405_BamHI_F	*secDF*	TATGGATCCGCAAAGCGTGGTACGAGAATTG	pTTQ18
Bc4405_XhoI_R	*secDF*	TATCTCGAGTACTTGCGGTTCAGATTCTACTTTC	pTTQ18
Bc4405_BamHI_F	*secDF*	ATTGGATCCATGGCAAAGCGTGGTACGAGAATTG	pHT304-pXyl
Bc4405_KpnI_R	*secDF*	ATAGGTACCTTA*GTG GTGATGGTGATGATG*TAC TTGCGGTTCAGATTCTACTTTC	pHT304-pXyl
Quantitative real-time PCR			*Product size [bp]*
Bc4405_qPCR_F	*secDF*	GATTGTAGTTGTAATTGCGGGTG	119
Bc4405_qPCR_R		TCAGACACAGTAACGGCTTG	
Bc1004_qPCR_F	*sigB*	GGGGCATTCACGTTCCAAGG	95
Bc1004_qPCR_R		CCTCGGTGAACGTTGTAGGTG	
Bc3522_qPCR_F	Bc3522	GAGAGCGTGGCTACGAAGGT	146
Bc3522_qPCR_R		GGGTAGCTCATTAGCAAGACCA	
Bc3522_qPCR_F	*hlyII*	TCCATAAAGCTGCCCCAGTT	118
Bc3522_qPCR_R		GCATCGGCATTTGGCCCTTT	
Bc5329_qPCR_F	*entA*	AGGCTCAAGCACCTGTAGCA	132
Bc5329_qPCR_R		TTTCGCTCGGGTGAGCTGTA	

Bc1991_qPCR_F	Bc1991	GTTCTGCCGTTTGCCAAGGA	113
Bc1991_qPCR_R		ATTCCACGCGTGAGCTTGTC	
Bc2271_qPCR_F	Bc2271	TTTTGGGTGGCGGACGTAGA	96
Bc2271_qPCR_R		TTTTTCCGCACCTAAACGGCG	
Bc2119_qPCR_F	Bc2119	AAAGCGGCACAACAGTCACC	121
Bc2119_qPCR_R		TAATTGGGCTAAGCGGCGGG	
Bc1436_qPCR_F	*pspA*	TGGCACATGCAAATCGTCGT	138
Bc1436_qPCR_R		ACGTGTCACGCTCATGCTCT	
Bc0862_qPCR_F	*proI*	GCCCTCTTCATACCGGGTGG	128
Bc0862_qPCR_R		AGTAGCTGAGGTCCGTGACA	
Bc5361_qPCR_F	Bc5361	AGGGGAGACAAGTTACGAGGC	121
Bc5361_qPCR_R		CCCTCGTTTTGCATCTGTCTCA	
Bc3826_qPCR_F	*codY*	TGGCGGTGAGCGTCTAGGTA	148
Bc3826_qPCR_R		ACGCGCTTCCTCTTCGATTT	
Bc4672_qPCR_F	*ccpA*	CGCGTTGTGAACGGTAACCC	106
Bc4672_qPCR_R		CTAGTCCACGTGCTACCGCA	
Bc5350_qPCR_F	*plcR*	GCACGCAGAAAAATTAGGAAGTG	129
Bc5350_qPCR_R		GGGTATACCGCACCCGATTC	
Bc1656_qPCR_F	*flag*	TGACGGTCAAAACCGCCCTA	125
Bc1656_qPCR_R		CCGCGCTTGCTTCTTGTTCT	
pBc0006_qPCR_F	*lexA*	TTGGTTTTGCCCCGTCTGTG	138
pBc0006_qPCR_R		ACAGTTGGATTGCTCGCGGT	
pBc0007_qPCR_F	pBC0007	CAAGCCGGAACCAATACCGC	120
pBc0007_qPCR_R		CTGACGTTCGGCTGCCTTTG	
Bc1110_qPCR_F	*cytK*	GCGCTGATAAACAGATTGCCGT	*
Bc1110_qPCR_R		GAAGCTTTAACAGAGCCACCA	
Bc1810_qPCR_F	*nheB*	CAATGGTACACAATGGGATCA	115
Bc1810_qPCR_R		TCCAGCTATCTTTCGCAATG	

Bc3102_qPCR_F<br>Bc3102_qPCR_R	*hlbB*	CTTGTACGATTCTACCAGGTA<br>GAGAATGAAAGAGACCTTGCA	144
16S_F<br>16S_R	*16S*	GGAAACCGGGGCTAATACC<br>GAGCCGTTACCTCACCAACT	121
Bc4306_qPCR_F<br>Bc4306_qPCR_R	*gatB*	AGCTGGTCGTGAAGACCTTG<br>CGGCATAACAGCAGTCATCA	175**
Plasmids used	*Relevant characteristics*		*Reference/Source*
pHT304-pXyl	E.coli / *Bacillus* shuttle vector, low-copy, ampr, eryr, xylose inducible promotor, Amp^r, Ery^r		D. Lereclus
pTTQ18n	High-copy E. coli protein overexpression vector, with C-terminal 6x His-tag, Amp^r		P. Henderson
pBKJ236	Temperature sensitive suicide vector carrying the I-SceI restriction site, Ery^r		[1]
pBKJ223	Tet^r, encoding the Isce-I homing endonuclease		[1]
Strains used	*Relevant characteristics*		
B. cereus ATCC 14579	Type strain, carrying pBClin15, AH1448		This laboratory
B. cereus ATCC 14579 ΔsecDF	Markerless knock-out of SecDF (Bc4405) in the B.cereus type strain,<br>Carrying pBClin15, AH1753		This study
E. coli BW25513 ΔacrB	Strain with a markerless deletion of acrB		K.M. Pos

*from Ceuppens *et al*. [2]; ** from Reiter *et al.* [3]

MIQE checklist for validation of microarray experiments

<u>Experimental design</u>: Likewise the microarray study *B. cereus* ATCC 14579 was defined as the control, while the isogenic Δ*secDF* mutant was addressed as the experimental strain. Two biological replicates (independent colonies) of each strain grown on the same plate were tested under identical conditions. Relative expression was determined as the fold change between the wild type and mutant strain, after normalization to the WT expression at time point 1h. The assay was carried out and analyzed in the principal investigators laboratory by AV.

<u>Samples</u>*:* Two independent cultures of both wild type and Δ*secDF* mutant strains were grown on LBG. Five ml cultures were harvested after 1h, 2h, 3h and 4h growth at 30 °C and incubated shortly in ice-cold methanol. After centrifugation, the cell pellets were stored at -20 °C until further usage.

RNA extraction: Isolation of RNA was done as previously described by Reiter *et al.* [3]. RNA samples were stored at -20 °C for no longer than 5 months. Upon usage the quality was controlled by UV-spectroscopy and gel electrophoresis and the RNA photometrical quantified using Biophotometer (Eppendorf).

Reverse transcription: cDNA synthesis was carried out using the SuperScript™ II Reverse Transcriptase (Invitrogen) as follows: 2µg total RNA were incubated with 2µl 50µM random hexamers (Applied Biosystems) and 2µl dNTP mix (10mM each, Invitrogen) at 65 °C for 5min. After cooling on ice, a mixture containing 8µl 5x First-Strand Buffer, 4µl 0.1M DTT, 40U Superase Inhibitor (Ambion) and 400U Reverse Transcriptase was added to give a final volume of 40µl. The reaction cycle included first a 10min incubation step at 25 °C followed by cDNA synthesis at 42 °C for 50min. The reaction was inactivated by heating to 70 °C for 15min. The absence of chromosomal DNA was controlled by amplifying *gatB* from reverse transcriptase-negative samples. If products were detected, none of those had a Cq-value below 34 (except WT sample 2 at t3: Cq 29), thus indicating no or trace amounts of chromosomal DNA contamination. The cDNA was stored at -20 °C.

qPCR target information: For validation 19 genes were selected based on their fold change values from the microarray experiments. Genes showing no, moderate and high difference were chosen as well as selected gene expression regulators. The oligonucleotides were constructed with primer blast, using default options and a product size of 90-130bp. Sequences (Table S6) were blasted against the non-redundant nucleotide database of *B. cereus* ATCC14579 and the annealing temperature was calculated using Oligocalc [4] nearest neighbour method. Salt-purified oligonucleotides were manufactured by Invitrogen.

qPCR protocol: 20µl qPCR reactions contained 2µl of 1:4 diluted cDNA (2µl of 1:400 dilution for 16S cDNA) template, 1µl each of oligonucleotides (0.25µM final concentration each) and 18µl of LightCycler® 480 SYBR Green I Master mix containing FastStart Taq DNA Polymerase, specifically made for the LightCycler® 480 Instrument (Roche). The assay was manually set up and conducted in 96-well plates from BIOplastics (B17489). The applied cycling parameters were as follows: 5min initial polymerase activation at 95C; followed by 45 cycles of 10sec denaturation at 95 °C, 15sec annealing at 57 °C and 10sec elongation at 72 °C. All samples were amplified in duplicates.

qPCR validation: Optimal oligonucleotide annealing temperatures were determined by gradient PCR using a temperature range of 49-60 °C. The specificity of the qRT-PCR products was evaluated by establishing the melting temperatures using the "Tm calling" function of the qPCR instrument. Control reactions without cDNA consistently gave Cq-values above 40.

PCR efficiencies were assessed once by diluting the pooled cDNA in 10-fold steps. Individual Cq values were plotted against the logarithm of the dilution factor and the slope was obtained the lines-of-best-fit from the Microsoft Office Excel software 2010. PCR efficiencies were calculated according to the formula $E = 10^{(-1/slope)}$. PCR efficiencies ranged from 1.7 to 2.0 with a fit of at least 0.98.

Data Analysis: The analysis modus "Abs Quant/2nd derivative Max" of the LightCycler® 480 analysis software 15.0.39 SP3 was used throughout the study to determine the expression

levels (Cq values). As reference genes 16S and *gatB* were selected. The high amount of ribosomal RNA is not expected to change throughout the experiments time frame ranging from early-exponential to early-stationary phase. To minimize mistakes introduced by the different dilution factors, a second gene, *gatB*, was chosen. This gene has been shown to be one of the most stable in the *B. cereus* type strain when grown in sporulation medium over the whole live cycle [3].

Following Vandesompele *et al.* [5] and using the previously freely available excel spread sheet form (http://medgen.ugent.be/~jvdesomp/genorm/), the expression level of each sample was calculated using a normalization factor based on the geometric mean of both reference genes. Each calculation contained 4 time points, where time point 1h of the wild type strain was set to expression level 1 and all the other time points of both wild type and mutant strain were accordingly rescaled. Fold differences were obtained by dividing the rescaled and normalized Cq values of the Δ*secDF* mutant by those of the wild type at the respective time points. For statistical analysis the built-in two-tailed, paired Student`s t-test formula of the MS Office Excel software 2010 was used.

References:

1. Janes BK, Stibitz S (2006) Routine Markerless Gene Replacement in Bacillus anthracis. Infection and Immunity 74: 1949-1953.
2. Ceuppens S, Timmery S, Mahillon J, Uyttendaele M, Boon N (2013) Small Bacillus cereus ATCC 14579 subpopulations are responsible for cytotoxin K production. J Appl Microbiol 114: 899-906.
3. Reiter L, Kolstø A-B, Piehler AP (2011) Reference genes for quantitative, reverse-transcription PCR in Bacillus cereus group strains throughout the bacterial life cycle. J Microbiol Methods 86: 210-217.
4. Kibbe WA (2007) OligoCalc: an online oligonucleotide properties calculator. Nucleic Acids Research 35: W43-W46.
5. Vandesompele J, De Preter K, Pattyn F, Poppe B, Van Roy N, et al. (2002) Accurate normalization of real-time quantitative RT-PCR data by geometric averaging of multiple internal control genes. Genome Biology 3: research0034.0031 - research0034.0011.
